# Simultaneous detection of miRNA and mRNA at the single‐cell level in plant tissues

**DOI:** 10.1111/pbi.13931

**Published:** 2022-10-20

**Authors:** Chi‐Chih Wu, Kun‐Ting Hsieh, Su‐Ying Yeh, Yen‐Ting Lu, Liang‐Jwu Chen, Maurice S. B. Ku, Wen‐Hsiung Li

**Affiliations:** ^1^ Biodiversity Research Center Academia Sinica Taipei Taiwan; ^2^ Institute of Molecular Biology, National Chung Hsing University Taichung Taiwan; ^3^ Department of Agricultural Biotechnology National Chiayi University Chaiyi Taiwan; ^4^ School of Biological Sciences Washington State University Pullman WA USA; ^5^ Department of Ecology and Evolution University of Chicago Chicago IL USA

**Keywords:** *in situ* detection, microRNA, LNA probe, padlock probe, Kranz anatomy

## Abstract

Detecting the simultaneous presence of a microRNA (miRNA) and a mRNA in a specific tissue can provide support for the prediction that the miRNA regulates the mRNA. Although two such methods have been developed for mammalian tissues, they have a low signal‐noise ratio and/or poor resolution at the single‐cell level. To overcome these drawbacks, we develop a method that uses sequence‐specific miRNA‐locked nucleic acid (LNA) and mRNA‐LNA probes. Moreover, it augments the detection signal by rolling circle amplification, achieving a high signal‐noise ratio at the single‐cell level. Dot signals are counted for determining the expression levels of mRNA and miRNA molecules in specific cells. We show a high sequence specificity of our miRNA‐LNA probe, revealing that it can discriminate single‐base mismatches. Numerical quantification by our method is tested in transgenic rice lines with different gene expression levels. We conduct several applications. First, the spatial expression profiling of osa‐miR156 and *OsSPL12* in rice leaves reveals their specific expression in mesophyll cells. Second, studying rice and its mutant lines with our method reveals opposite expression patterns of miRNA and its target mRNA in tissues. Third, the dynamic expression profiles of *ZmGRF8* and zma‐miR396 during maize leaf development provide evidence that zma‐miR396 regulates the preferential spatial expression of *ZmGRF8* in bundle sheath cells. Finally, our method can be scaled up to simultaneously detect multiple miRNAs and mRNAs in a tissue. Thus, it is a sensitive and versatile technique for studying miRNA regulation of plant tissue development.

## Graphical abstract

A method is developed for simultaneous detection of microRNAs and mRNAs in plant tissues, achieving a high signal‐noise ratio at the single‐cell level. Dot signals are counted for determining the expression levels of microRNA and mRNA molecules in specific cells.

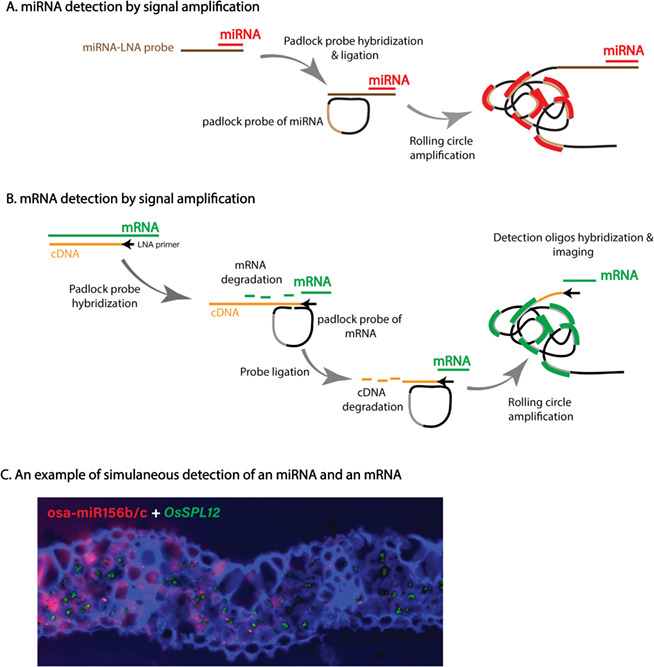

## Introduction

It is well known that microRNAs (miRNAs) participate in the regulation of various developmental processes through post‐transcriptional gene silencing. Although miRNAs have been extensively studied, there is still often the need to determine the simultaneous presence of a miRNA and its target mRNA in a specific tissue. Two methods have been developed for mammalian tissues for this purpose (Kasai *et al*., 2016; Zhuang *et al*., 2020), but both of them have some drawbacks. First, the locked‐nucleic‐acid (LNA) probe approach, used regularly in *in situ* hybridization, has a low signal‐to‐noise ratio (Kasai *et al*., 2016), so that the real signals are not obviously distinguishable from autofluorescence, such as in plant materials (Huang *et al*., 2019). Second, the other method, which is based on *in situ* hybridization chain reaction, generally results in overwhelming signals (Zhuang *et al*., 2020), so it is difficult to identify signals at the resolution of single cells for numerical quantification.

In this study, we have developed a method for simultaneously studying the spatial–temporal expression patterns of miRNAs and mRNAs in plant tissues to achieve resolution at the single‐cell level. To detect mRNA transcripts in various cell types, we have previously modified a histologically explicit padlock‐probe assay coupled with rolling circle amplification (RCA) that allows the detection of RNA transcripts in animal tissues (Wu *et al*., 2019). After the RCA amplification, aggregation of repeated single‐strand oligonucleotides forms the rolling circle product (RCP), which can be hybridized to a specific fluorescence‐tagged detection oligonucleotide to produce bright‐dot signals of different fluorescence colours. Compared to other methods of *in situ* hybridization, the padlock‐probe assay with RCA provides a higher signal‐noise ratio (Weibrecht *et al*., 2013; Wu *et al*., 2019), greatly reducing the interference from the common strong fluorescence background derived from plant tissues. Dot signals can be counted for estimating relative expression levels of mRNA molecules in specific tissues or cells. Furthermore, hybridization of the LNA primer to mRNA molecules provides a high specificity to detect target genes (Latorra *et al*., 2003).

Since functional miRNAs are only 20–21 bases long, a miRNA molecule cannot serve as a template for reverse transcription as does an mRNA molecule. Therefore, we develop a new technique by taking the advantage of LNA probe and the padlock probe with RCA to study the expression pattern of a miRNA in plant tissues. Combining this technique with the padlock probe technique for detecting mRNAs, one can simultaneously detect miRNA and mRNA molecules on the same tissue section for numerical quantification. We tested the sequence specificity of miRNA‐LNA probes using modified dot blot and transformed protoplasts with various point mutations of miRNAs, revealing that our miRNA‐LNA probe is capable of single‐base‐mismatch discrimination. Furthermore, by using transgenic rice lines that show various expression levels of miRNAs and mRNA, we demonstrated that our method is capable of numerical quantification for both miRNA and mRNA molecules.

Maize embryonic leaf is a model system for studying the cellular development and the genetic control network of Kranz anatomy development in C_4_ leaves (Chang *et al*., 2012, 2019; Liu *et al*., 2013; Yu *et al*., 2015). MicroRNA miR396 of *Arabidopsis* has been shown to regulate leaf size and vegetative transition (Hou *et al*., 2019), and the zma‐miR396‐growth regulating factor (GRF) network is associated with maize grain morphogenesis (Zhang *et al*., 2015). Moreover, maize miR396 has been shown to regulate maize *GRF8* in developing maize ears identified by combining small RNA and degradome sequencing (Liu *et al*., 2014). Since GRF genes are wildly expressed during maize leaf development (Chang *et al*., 2012, 2019), we use our new method to examine the expression profiles of zma‐miR396 and its predicted target *ZmGRF8* in developing maize leaves, providing evidence that zma‐miR396 indeed regulates the expression of *ZmGRF8* in a cell‐specific manner during maize leaf development.

## Results

### Design of miRNA‐LNA probes for *in situ* detection of miRNA molecules

**Figure 1:**
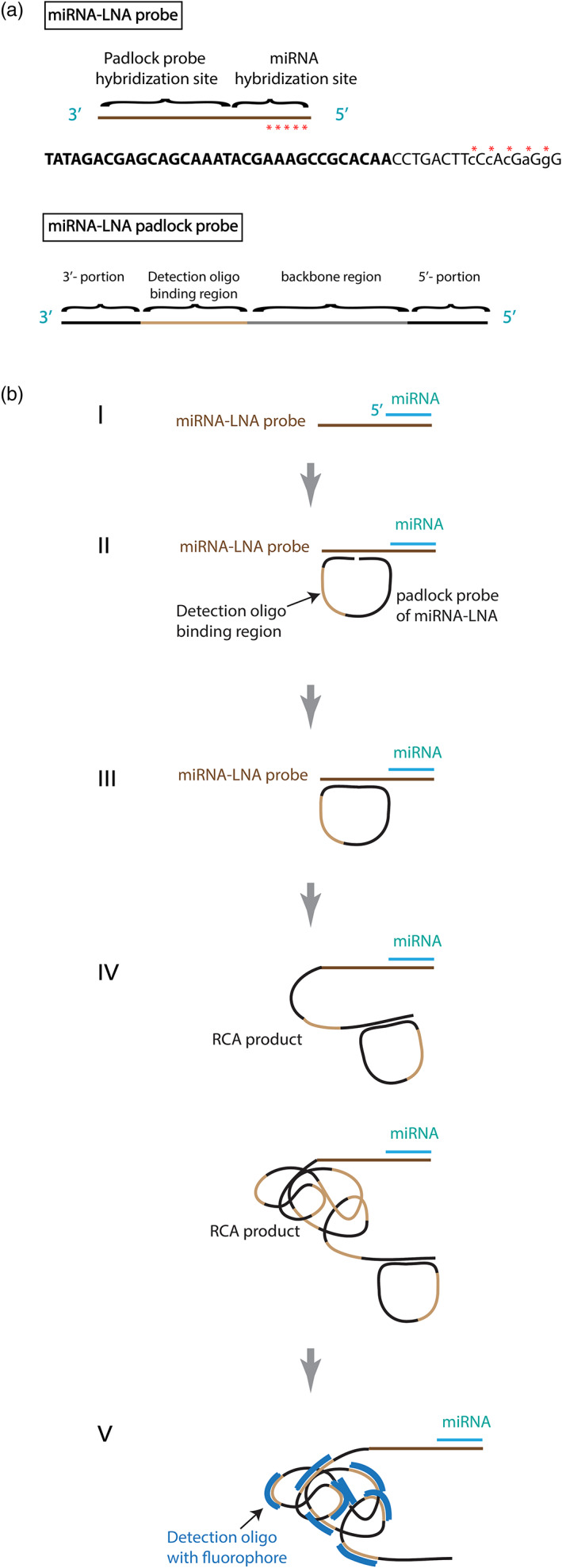
A method for detecting miRNA molecules on a plant section via a LNA probe and a padlock probe with rolling circle amplification (RCA). (a) The design of a miRNA‐LNA probe and a miRNA‐LNA padlock probe. The miRNA‐LNA probe comprises a miRNA hybridization site (the same length as the target miRNA size, *e.g*., 20 bases) at 5′ end and a padlock probe hybridization site of 30 bases long at the 3′ end. The five red stars indicate the LNA modified bases at the 2nd, 4th, 6th, 8th, and 10th bases from the 5′end. For instance, the sequence of zma‐miR319b‐3p LNA probe is listed underneath the drawing of the miRNA‐LNA probe, in which the bases in the lowercase with red stars are LNA modified nucleotides, and the bases in bold are the padlock probe hybridization site. The miRNA‐LNA padlock probe consists of four regions: the 5′‐ and 3′‐ portions (in black) complementary to the padlock probe hybridization site of the miRNA‐LNA probe, the detection oligo binding region (in light brown) and the backbone region (in grey). (b) The five steps of the miRNA detection method. I. The hybridization of a targeted miRNA (light blue, *e.g*., 20 bases) with the 5′ first 20 bases of a miRNA‐LNA probe (dark brown). II. The hybridization of the two padlock probe ends (black) to the “padlock probe hybridization site” of the miRNA‐LNA probe, which is the remaining 30 free bases of the miRNA‐LNA probe. The padlock probe contains a unique 20‐base sequence of “detection oligo binding site” (light brown). III. The ligation between the 5′‐ and 3′‐ends of the padlock probe. When the 15 bases of the 5′ and 3′ ends of the padlock probe perfectly hybridize to the “padlock probe hybridization site” of the miRNA‐LNA probe, the ligase would fill the gap between 5′ and 3′ ends to form a circled padlock probe. IV. The signal amplification by the RCA. Phi29 polymerase uses the circled padlock probe as the template to produce a long single‐strand RCA product (black + light brown). V. Hybridization of fluorescence detection probes (dark blue) to the “detection oligo binding regions” (light brown) of the RCA product.

To achieve signal quantification at the single‐cell level, our miRNA‐LNA probe design adopts the rolling circle amplification (RCA). The miRNA‐LNA probe is of 50 bases, in which the first 20 bases at the 5′‐end perfectly match the miRNA sequence, which generally is 20 bases long (Figure 1a). There are five LNA modified sites at the 2nd, 4th, 6th, 8th and 10th positions from the 5′ end, forming a 5′ LNA modification region, which increases both the binding strength of the probe and nucleotide mismatch discrimination. The remaining part consists of a unique sequence of 30 bases that are not or rarely present in RNA transcripts of the target species (here maize or rice), so it can serve as a template for hybridization (the padlock probe hybridization site) with a designed padlock probe (Figure 1a). The padlock probe is an 80‐base 5′‐phosphorylated oligonucleotide consisting of a 15‐base sequence at the 5′end (5′‐portion) and another at the 3′end (3′‐portion) for hybridization to the 30‐base padlock probe hybridization site of the miRNA‐LNA probe, and the remaining part consists of a unique 20‐base detection oligo binding site and a 30‐base backbone fragment (Figure 1a). The sequences of 5′‐portion and 3′‐portion of the padlock probe match the two halves of the padlock probe hybridization site of the miRNA‐LNA probe (Figure 1a).

Our detection procedure comprises five major steps (Figure 1b): (i) the hybridization of miRNA‐LNA probe to miRNA molecule, (ii) the hybridization of padlock probe to a miRNA‐LNA probe coupled with a miRNA, (iii) the ligation of the gap between 5′‐ and 3′‐ ends of hybridized padlock probe, (iv) the rolling circle amplification of the annealed circle padlock probe, and (v) the detection oligonucleotide hybridization. In the first step (Figure 1b‐I), the designed miRNA‐LNA probe is hybridized to target miRNA molecules fixed on tissue sections. When the 5′ and 3′ends of the padlock probe perfectly hybridize to the free 3′end of the miRNA‐LNA probe (Figure 1b‐II), ligation of the gap occurs (Figure 1b‐III) and a RCA product is produced using phi29 DNA polymerase (Figure 1b‐IV). The amplified sequences can be detected by detection oligonucleotides tagged with fluorescence markers (Figure 1b‐V).

### *In vitro* test of the probe design and the binding specificity of miRNA‐LNA probe

**Figure 2:**
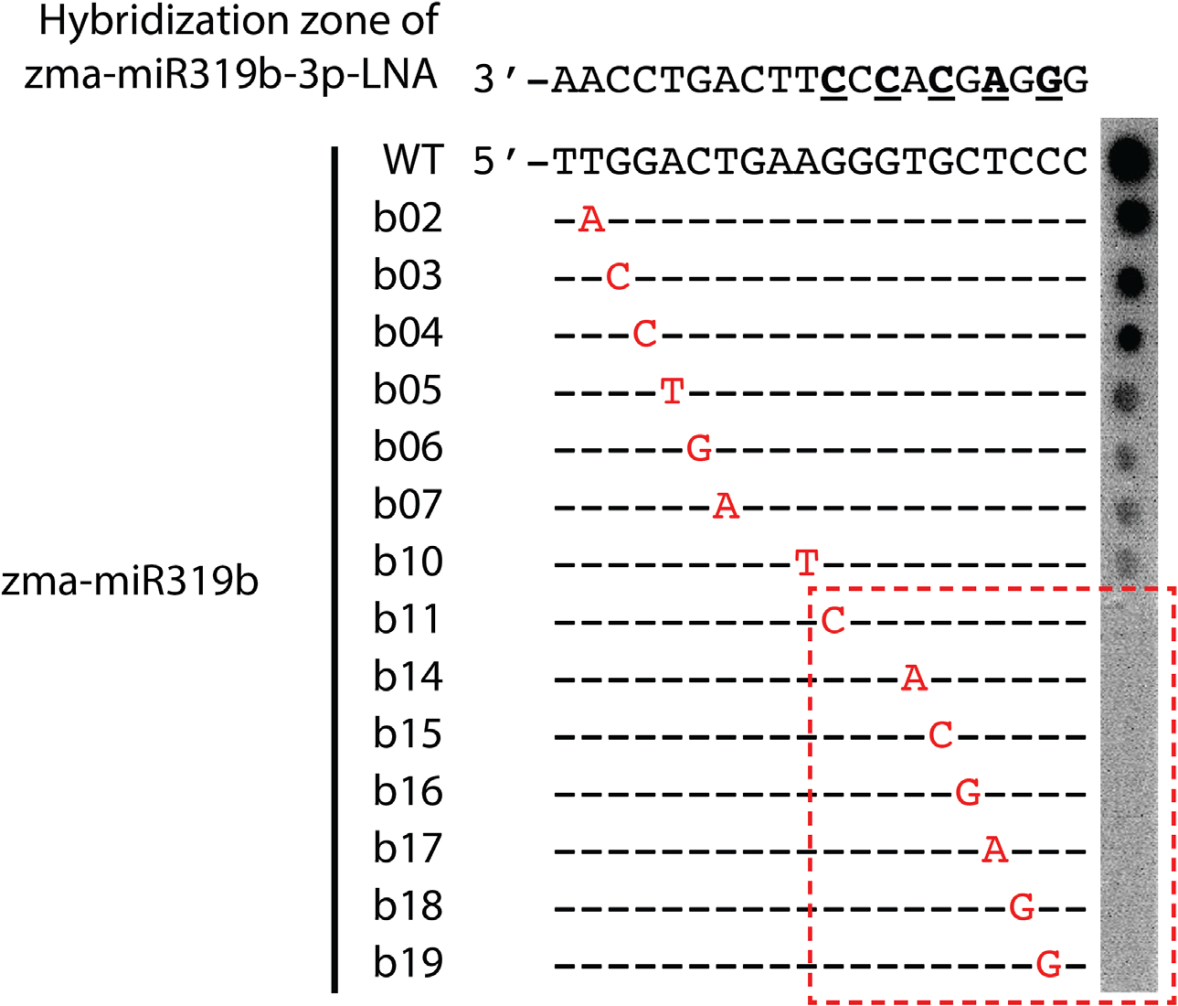
Modified dot blot hybridization showing the effect of a single nucleotide mismatch between the zma‐miR319b‐LNA probe and a target sequence on the binding between the probe and the target sequence. Only the 20‐base sequence of the hybridization zone of the zma‐miR319b‐LNA probe is shown. The bases in bold with an underline indicate locked‐nucleic acid modified monomers at the 2nd, 4th, 6th, 8th and 10th position from the 5′ end. The target sequence is synthesized as deoxyribonucleotides, representing a target miRNA molecule. Each base in red is a mismatch to the zma‐miR319b‐WT sequence, which represents zma‐miR319b‐3p. The suffix b02 represents a sequence with a mismatch at the second position of zma‐miR319b‐3p, b03 represents a mismatch at the third position and so on. The red rectangle indicates the portions of the target sequences corresponding to the 5′ LNA modification region of the zma‐miR319b‐LNA probe. The signal intensity (the right‐hand bar) decreases from a target sequence with the mismatched site at the 2nd to 10th base and no signal is seen for a target sequence with the mismatched site within the 11th and the 19th base on the dot blot hybridization.

To verify our new detection design and to examine the binding specificity of our miRNA‐LNA probe, we use zma‐miR319b‐3p, which is 20 bases long, as an example to test the detection protocol and the effect of a single mismatch between the target sequence and the miRNA‐LNA probe on the binding affinity (see the mismatch layout in Figure 2). For these purposes, we first modify the protocol of dot blot to demonstrate our protocol *in vitro* and evaluate the binding affinity between the zma‐miR319b‐LNA probe and synthesized target DNA sequences each with a mismatch in between the 2nd and the 19th base (Figure 2). The hybridization affinity of the LNA probe to a target sequence is measured by bioluminescent intensity and the hybridization temperature is set 10 °C below the predicted melting temperature of the zma‐miR319b‐LNA probe. The sequence denoted by zma‐miR319b‐WT, which represents the wild‐type zma‐miR319b‐3p, is used as the reference, and it shows the most intense signals, suggesting that our protocol works properly (Figure 2). The signal of dot blotting gradually decreases from the zma‐miR319b‐b02 to zma‐miR319b‐b10 (point mutants of zma‐miR319b‐3p), which are complementary to the region outside of the LNA modification region of miRNA‐LNA probe (Figure 2). The signal becomes invisible when the mismatch is at the 11th position and so on until the 19th position, which are within the LNA modification region of the miRNA‐LNA probe (Figure 2). By the modified dot blot, we verify that our miRNA‐LNA probe design and detection protocol work well for detecting synthesized target sequences (e.g., zma‐miR319b‐WT). Furthermore, the effect of a single mismatch on binding affinity is stronger when the mismatch is at or near the 3′ end of the target sequence, which corresponds to the 5′ LNA modification region of the zma‐miR319b‐LNA probe than it is at or near the 5′ end. In conclusion, our miRNA‐LNA probe has a high sequence specificity *in vitro* (Figure 2).

### Testing the probe design and binding specificity of miRNA‐LNA probe on protoplasts and *in situ* visualization on plant sections

We test for detection on isolated maize mesophyll protoplasts and *in situ* detection on tissue sections to evaluate the performance of our miRNA‐LNA probe. Based on the signal intensity by the modified dot blot (Figure 2), we construct over‐expression plasmids with the DNA fragment containing different single‐nucleotide‐mismatch versions of zma‐miR319b‐3p, including zma‐miR319b‐b03, zma‐miR319b‐b05, zma‐miR319b‐b11 and zma‐miR319b‐b19 (Figures 2 and S1). Each of these sequences has a single nucleotide mismatch in the hybridization region of the miRNA‐LNA probe and cover different intensities of bioluminescence within and outside of the LNA modification region (Figures 2 and S1). The plasmid with zma‐miR319b‐WT is used as a positive control, and the non‐transformed protoplast is a negative control. GFP signals of transformed protoplasts are checked to confirm successful transformation, and all designed versions of zma‐miR319b‐3p in transformed protoplasts are highly expressed, as analysed by qRT‐PCR (Figure S2).

**Figure 3:**
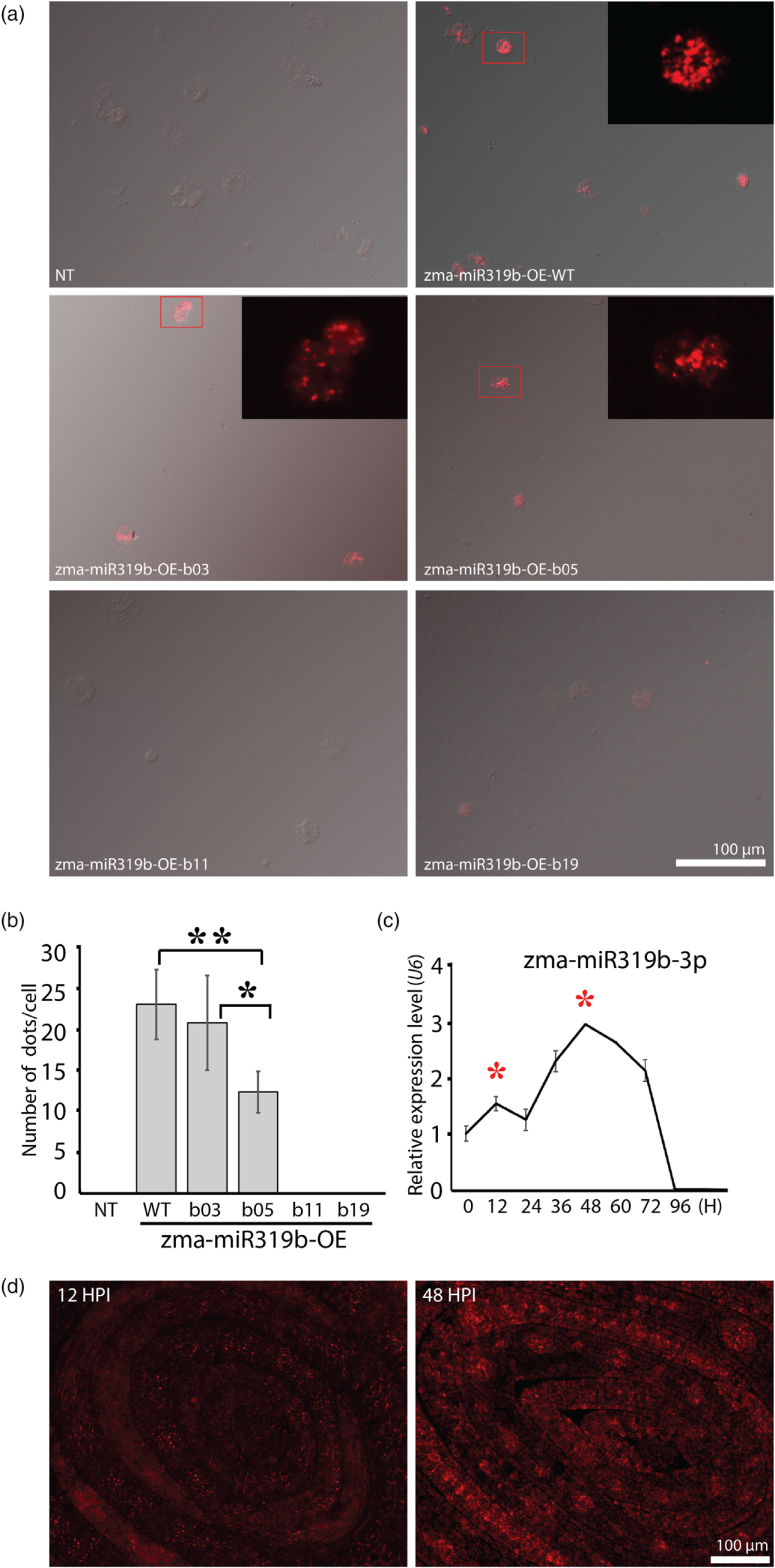
Detection of miRNA molecules by padlock probe signals on fixed transformed maize protoplasts and *in situ* detection on leaf sections. (a) Images showing RCA signals on maize non‐transformed (NT) protoplasts and protoplasts transformed with various zma‐miR319b overexpression vectors (denoted by WT, b03, b05, b11 and b19). Abundant signals are seen in protoplasts overexpressed with WT and b03 sequences, but no signal is visible in NT and in protoplasts with overexpressed b11 and b19 sequence. The protoplasts with b05 have a moderate level of signals. NT (non‐transformed) is the negative control. (b) Quantification of RCA signal dots on protoplasts transformed with a sequence carrying a single nucleotide mismatch to the zma‐miR319b‐WT. Mismatch sites are shown in Figure 2. The counts of signal dots per protoplast transformed with various zma‐miR319b overexpression vectors are consistent with the trend of signal intensity on modified dot blot hybridization in Figure 2 (*, *P* ~ 0.05; **, *P* < 0.01; *n* = 5). (c) Relative expression levels of zma‐miR319b‐3p of maize seedling at different time points after imbibition, as assayed by qRT‐PCR. The expression level changes with the time after imbibition and the highest expression level is at 48 h post imbibition (HPI). The two red stars indicate two developmental stages used for the *in situ* detection in (d). (d) *In situ* detection of zma‐miR319b‐3p on maize developing embryonic leaves at 12 and 48 HPI. Rolling circle products (RCPs) are round bright red dots, showing a high signal‐to‐noise (autofluorescence) ratio. Signals of zma‐miR319b‐3p are more abundant at 48 HPI than at 12 HPI, consistent with the expression trend assayed by qRT‐PCR (c).

We then perform our miRNA detection method (described in Figure 1b) on protoplasts fixed on slides. The results show that bright‐round‐dotted fluorescence rolling circle amplification (RCA) signals coupled with miRNA molecules are abundant on protoplasts transformed with zma‐miR319b‐WT, zma‐miR319b‐b03 and zma‐miR319b‐b05, but no signal is visible on protoplasts transformed with zma‐miR319b‐b11and zma‐miR319b‐b19, and on non‐transformed protoplasts as well (Figure 3a). Since, in general, each RCA dot represents a miRNA molecule, we can use the number of dots per protoplast as an indicator of expression level. By using the pipelines (Figure S3) of the CellProfiler (Carpenter *et al*., 2006), the quantification result shows that protoplasts of zma‐miR319b‐WT and zma‐miR319b‐b03 have a higher number of signals than that of zma‐miR319b‐b05, and no signal is detected on protoplasts transformed with zma‐miR319‐b11 and zma‐miR319‐b19 (Figure 3b), suggesting a high sequence specificity of our miRNA‐LNA probe. The specificity trend of these five versions of zma‐miR319b‐3p in terms of dot number is similar to the expression intensity trend by the above‐modified dot blot, confirming a high sequence specificity of our miRNA‐LNA probe in fixed protoplasts.

For *in situ* detection, we apply our technique to leaf sections of maize seedlings. miR319s are suggested to regulate a subset of the TEOSINTE BRANCHED1/CYCLOIDEA/PROLIFERATING CELL FACTOR (TCP) family and are required for petal and leaf development in plant species, but there is no confirmation data in maize (Fang *et al*., 2021). We, therefore, examine sections of embryonic leaves of maize seedlings 12 and 48 h post imbibition, because the relatively low expression level at 12 h and the highest expression level at 48 h post imbibition are found by qRT‐PCR (Figure 3c). After running our miRNA *in situ* detection, the images show that the amount of RCA‐dotted signals is higher on embryonic leaves at 48 h post imbibition than that at 12 h post imbibition (Figure 3d). In addition, zma‐miR319b‐3p signals are much stronger in developing vascular bundles and mesophyll cells near leaf margins than in other leaf regions (Figure 3d). Overall, our method detects miRNA molecules on fixed protoplasts (*semi‐in‐vivo*) and on leaf sections (*in‐situ*). In addition, our method can quantify target miRNA molecules.

### Simultaneous detection of miRNA and mRNA molecules in tissues

**Figure 4:**
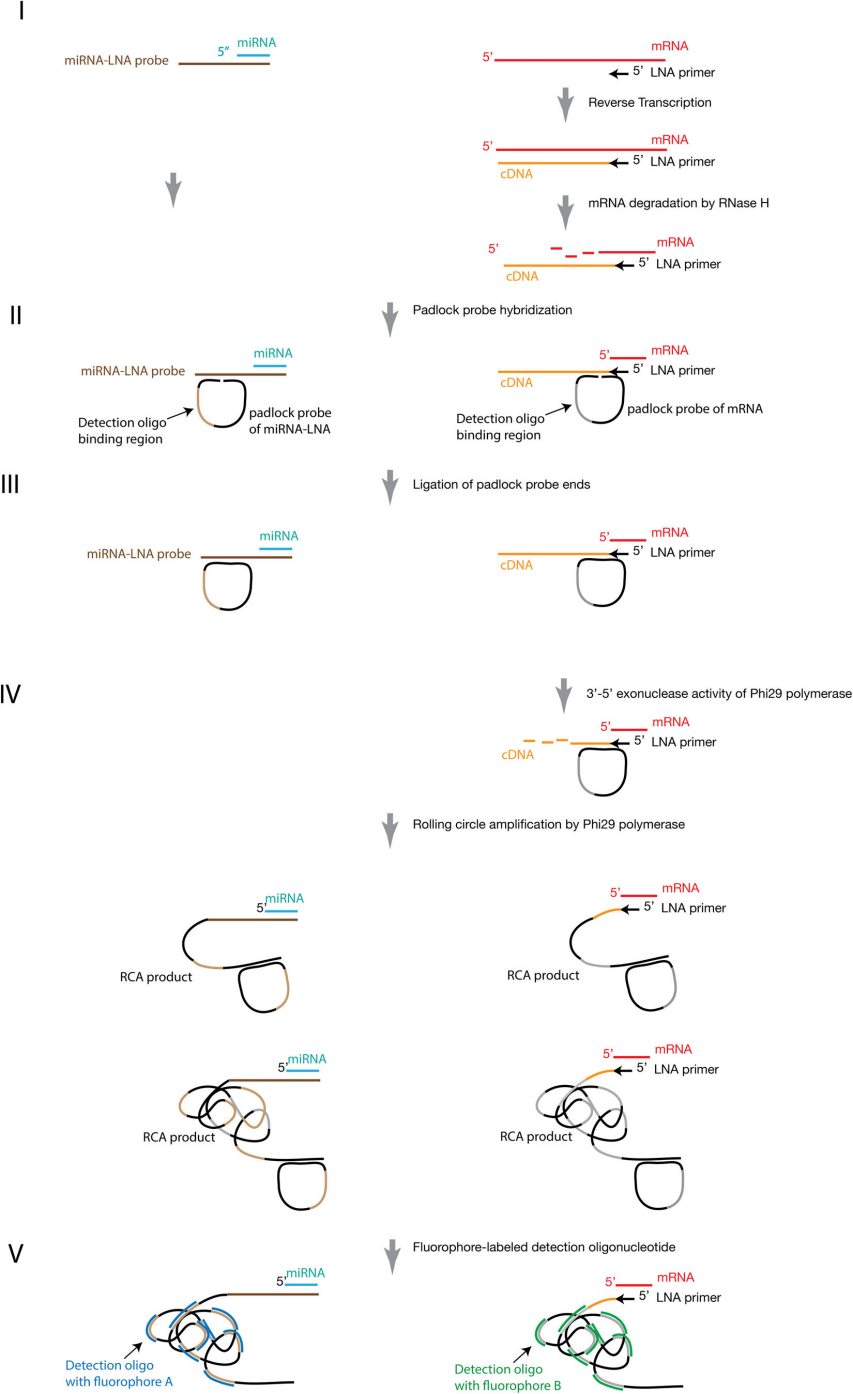
Simultaneous detection of miRNA and mRNA molecules in a plant tissue section. The schematic diagram shows the five steps (I–V) for miRNA (on the left‐hand side) and mRNA (on the right‐hand side) detection in parallel. (I) The hybridization of a miRNA‐LNA probe (dark brown) to a miRNA (light blue, on the left‐hand side) and the reverse transcription of the target mRNA transcript (red, on the right‐hand side) with the LNA primer (black). (II) The hybridization of the miRNA padlock probe to the miRNA‐LNA probe (the left‐hand side) and the hybridization of the mRNA padlock probe to the cDNA‐LNA primer‐mRNA complex (the right‐hand side). (III) The ligation between the 5′‐ and 3′‐ends of the two padlock probes. (IV) Single strand cDNA digestion and RCA products of both circled padlock probes. (V) The hybridization of unique detection oligonucleotides containing different fluorophores (dark blue for miRNA, green for mRNA) to the corresponding detection oligo binding sites of the two types of RCA products. Both fluorophores are visualized on the same tissue section.

By combining the above miRNA detection method and the mRNA detection method, we can simultaneously detect miRNA and mRNA molecules on the same plant tissue section. The procedure comprises five steps (Figure 4). First, the miRNA‐LNA probe is hybridized to the miRNA, and the mRNA‐LNA primer is hybridized to the mRNA, so that the cDNA sequence is produced from the reverse transcription of the mRNA transcript with the mRNA‐LNA primer (Figure 4‐I). Thus, sequence templates of padlock probe hybridization sites are available for both miRNA and mRNA padlock probes (Figure 4‐I). Second, the miRNA padlock probe containing a unique detection oligo binding site is hybridized to the miRNA‐LNA probe and mRNA padlock probe is hybridized to the cDNA‐LNA primer‐mRNA complex, respectively (Figure 4‐II). Third, the 5′‐ and 3′‐ends of padlock probes are ligated (Figure 4‐III). Fourth, the RCAs of both circled padlock probes are amplified by phi29 DNA polymerase using circled padlock probes to form long‐single‐strand nucleotides (Figure 4‐IV). Finally, the unique detection oligonucleotides containing different fluorophores are hybridized to the corresponding detection oligo binding sites of the two types of RCA products. Then, both fluorophores for miRNA and mRNA molecules can be simultaneously visualized on the same tissue section (Figure 4‐V).

### Simultaneous detection of miRNA and mRNA molecules in leaf tissues of rice and maize

To test our method on the simultaneous quantification of signals on tissues, we examine signals on plant leaves with different expression levels of miRNAs and target mRNAs. For this purpose, we first investigate osa‐miR156b and its targets, *OsSPLs* (Xie *et al*., 2006), on *miR156b/c* T‐DNA activation‐tagged mutants (*miR156b/c*_*Act*_; Hsing *et al*., 2007) and *miR156b/c* overexpression transgenic rice (*miR156b/c‐OE*). These plant materials allow us to examine the spatial expression patterns of osa‐miR156 and *OsSPLs*.

**Figure 5:**
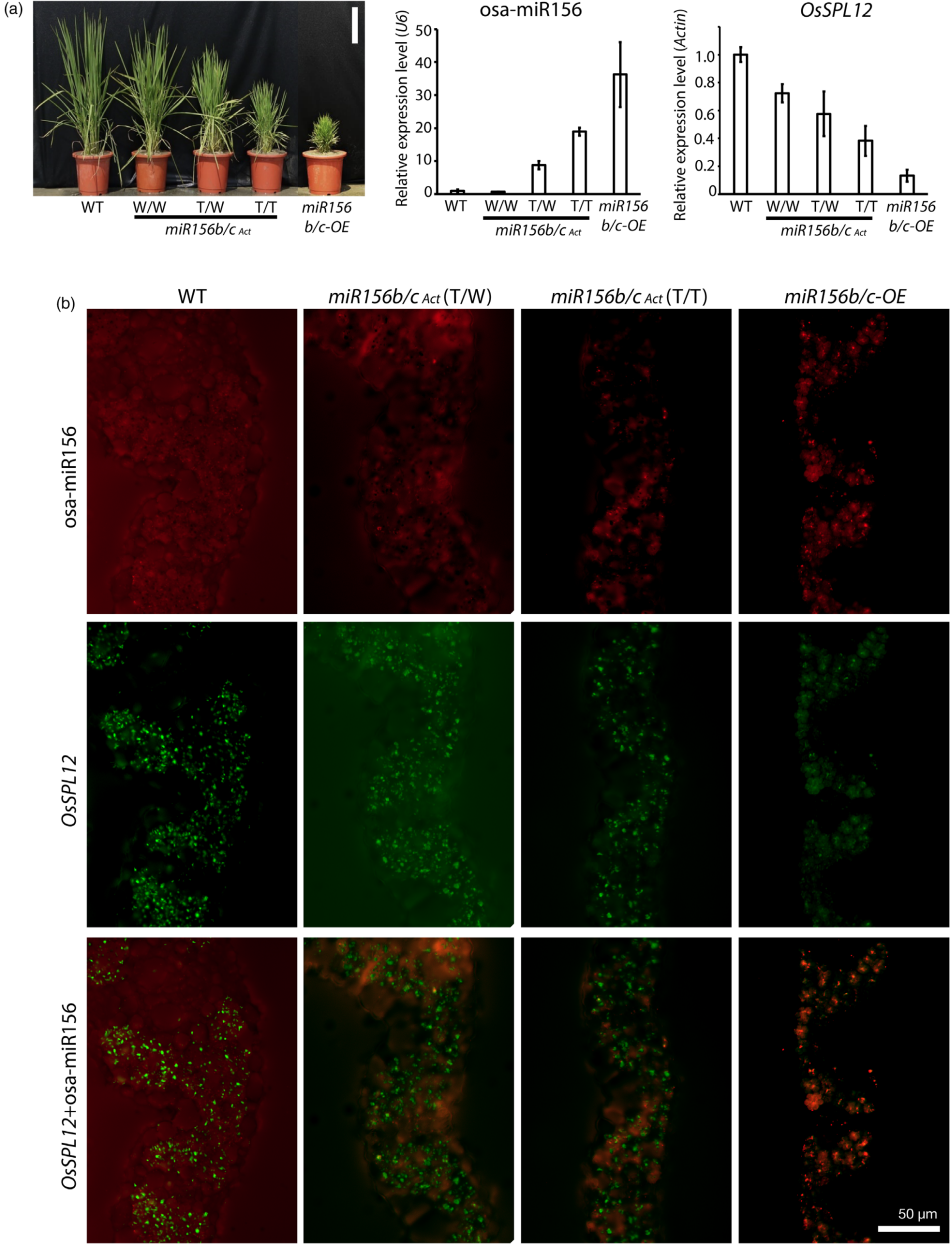
The relative expression levels of osa‐miR156 and *OsSPL12* in osa‐miR156b/c T‐DNA activation‐tagged mutants (*miR156b/c*_*Act*_) and osa‐miR156b/c overexpression transgenic rice (*miR156b/c‐OE*) and simultaneous *in situ* detection of osa‐miR156 and *OsSPL12* on leaf sections. (a) Phenotypes of WT, *miR156b/c*_*Act*_ mutants (W/W, segregated WT; T/W, heterozygous; T/T, homozygous) and *miR156b/c‐OE*. The relative expression levels of osa‐miR156 and *OsSPL12* are assayed by qRT‐PCR relative to a reference for *in situ* detection. Bar = 20 cm. (b) Simultaneous *in situ* detection of osa‐miR156 and *OsSPL12* on same leaf sections of WT, *miR156b*_*Act*_ mutants and *miR156b/c‐OE*. The three rows of images show *in situ* patterns of individual signals (osa‐miR156 or *OsSPL12*, the first two rows), and combined signals (*OsSPL12*+osa‐miR156, the third row) on the same section.

After genotyping, we obtain heterozygous *miR156b/c*_*Act*_ mutants (T/W), homozygous *miR156b/c*_*Act*_ mutants (T/T), and miR156b/c overexpression transgenic rice (*miR156b/c‐OE*), showing morphological changes (Figure 5a). The T‐DNA activation lines and the overexpression transgenic rice both show dwarfism phenotypes, compared to WT (Figure 5a). In WT, the expression level of osa‐miR156 changes as leaves develop (Figure S4). The youngest leaf has a relatively low expression level of osa‐miR156, compared to more mature leaves by both qRT‐PCR and *in situ* miRNA detection (Figure S4). In addition, *OsSPL12* has the highest relative expression level in the young leaf, compared to *OsSPL2*, *OsSPL11 and OsSPL13* (Figure S5). Thus, the youngest leaves are selected for the comparison of miR156b and *OsSPL12* expression profiles among rice lines of T‐DNA activation or overexpression.

By qRT‐PCR, the expression level of osa‐miR156 of the homozygous *miR156b/c*_*Act*_ mutants (T/T) is higher than that of heterozygous *miR156b/c*_*Act*_ mutants (T/W), and miR156b/c overexpression transgenic rice (*miR156b/c‐OE*) shows the highest expression level (Figure 5a). In contrast, *OsSPL12* shows a reverse expression profile (Figure 5a). Thus, we predict that osa‐miR156 and *OsSPL12* exhibit opposite expression patterns in the same tissue or cells over the developing trajectory.

The results of our simultaneous detection of osa‐miR156 and *OsSPL12* signals on rice leaf tissues are shown in Figures 5b and S6. The three rows show the data for miR156, for *OsSPL12* and for both miR156 and *OsSPL12* together, respectively. Note that *miR156b/c‐OE* has much more signals of osa‐miR156, and the signals become serially less in *miR156b/c*_*Act*_ (T/T), *miR156b/c*_*Act*_ (T/W), and WT (the first and third rows in Figures 5b and S6). In contrast, *OsSPL12* displays the reverse expression trend, in which WT has much more signals of *OsSPL12*, and the signals become weaker and weaker in *miR156b/c*_*Act*_ (T/W), *miR156b/c*_*Act*_ (T/T), and *miR156b/c‐OE* (the second and third rows in Figures 5b and S6). Thus, our method provides a tool for *in situ* numerical quantification with a wide range of signal intensities for both miRNAs and mRNA simultaneously. The trends of expression profiles of osa‐miR156b and *OsSPL12* are consistent with those by qRT‐PCR (Figure 5a). Moreover, based on the expression patterns on leaf tissues, we find both osa‐miR156 and *OsSPL12* expressed in rice mesophyll cells (Figures 5b, S3, S4 and S7).

**Figure 6:**
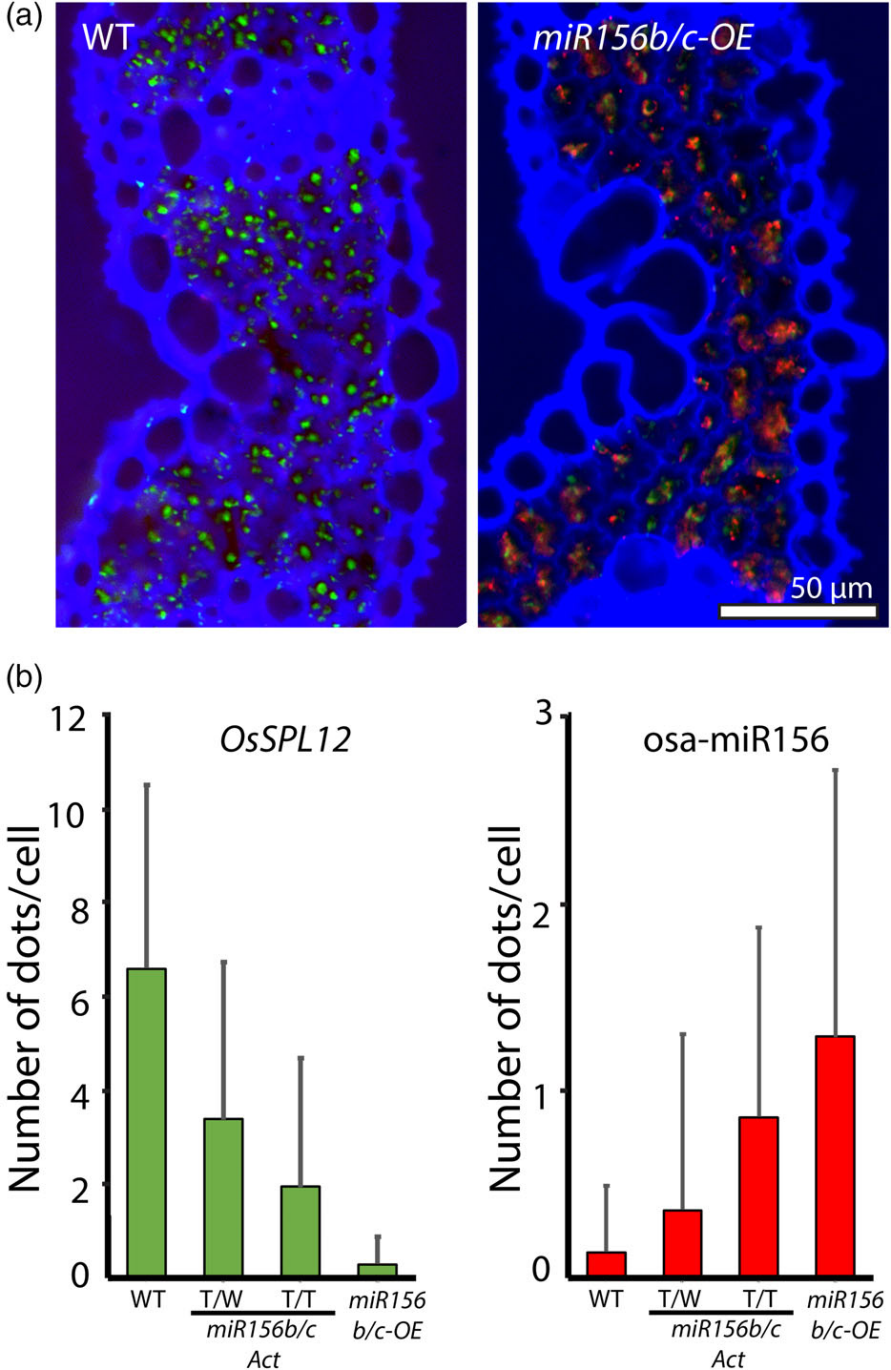
Numerical quantification of signal dots in cells of rice leaf by RCA signals. (a) Enhanced combined images showing RCA signals of *OsSPL12* (green dots) or osa‐miR156 (red dots) in WT or *miR156b/c‐OE*. WT has abundant *OsSPL12* signals, while *miR156b/c‐OE* has abundant osa‐miR156 signals in a cell. The blue signal is autofluorescence of cell wall, showing the cell boundary. Three individual channels of each enhanced image are used for dot signal counting (*OsSPL12* and osa‐miR156) and for the identification of cell boundary (blue autofluorescence). Bar = 50 μm. (b) Bar graphs showing the average number of dot signals per cell of *OsSPL12* and osa‐miR156 of WT, osa‐miR156b/c T‐DNA activation‐tagged mutants (*miR156b/c*_*Act*_) and osa‐miR156b/c overexpression transgenic rice (*miR156b/c‐OE*), indicating high heterogeneity of cells in term of dot counts. The trends of expression level of *OsSPL12* and osa‐miR156 in term of dot number per cell among these rice lines are consistent with those by qRT‐PCR (Figure 5a).

Since each RCA signal dot is derived from the conjugation of a padlock probe with a mRNA or a miRNA molecule, we count the mRNA or miRNA molecules in a cell by counting the RCA signals of *OsSPL12* and osa‐miR156 within individual mesophyll cells of leaf tissues. By adjusting the brightness and contrast of images, the autofluorescence of cell wall, the boundary of a cell, can be determined, and the signals of *OsSPL12* and osa‐miR156 within a cell can then be counted using the CellProfiler pipeline (Figures 6a and S7). The result shows that cells of leaves are highly heterogeneous in terms of dot counts of these two genes (Figure 6b). This high heterogeneity may be due to different physiological stages of cells, or non‐even distribution of miRNA or mRNA molecules in the cytosol. Overall, the trends of expression level of *OsSPL12* and osa‐miR156 in terms of the average dot number per cell among these rice lines (Figure 6b) is consistent with that by qRT‐PCR (Figure 5a).

**Figure 7:**
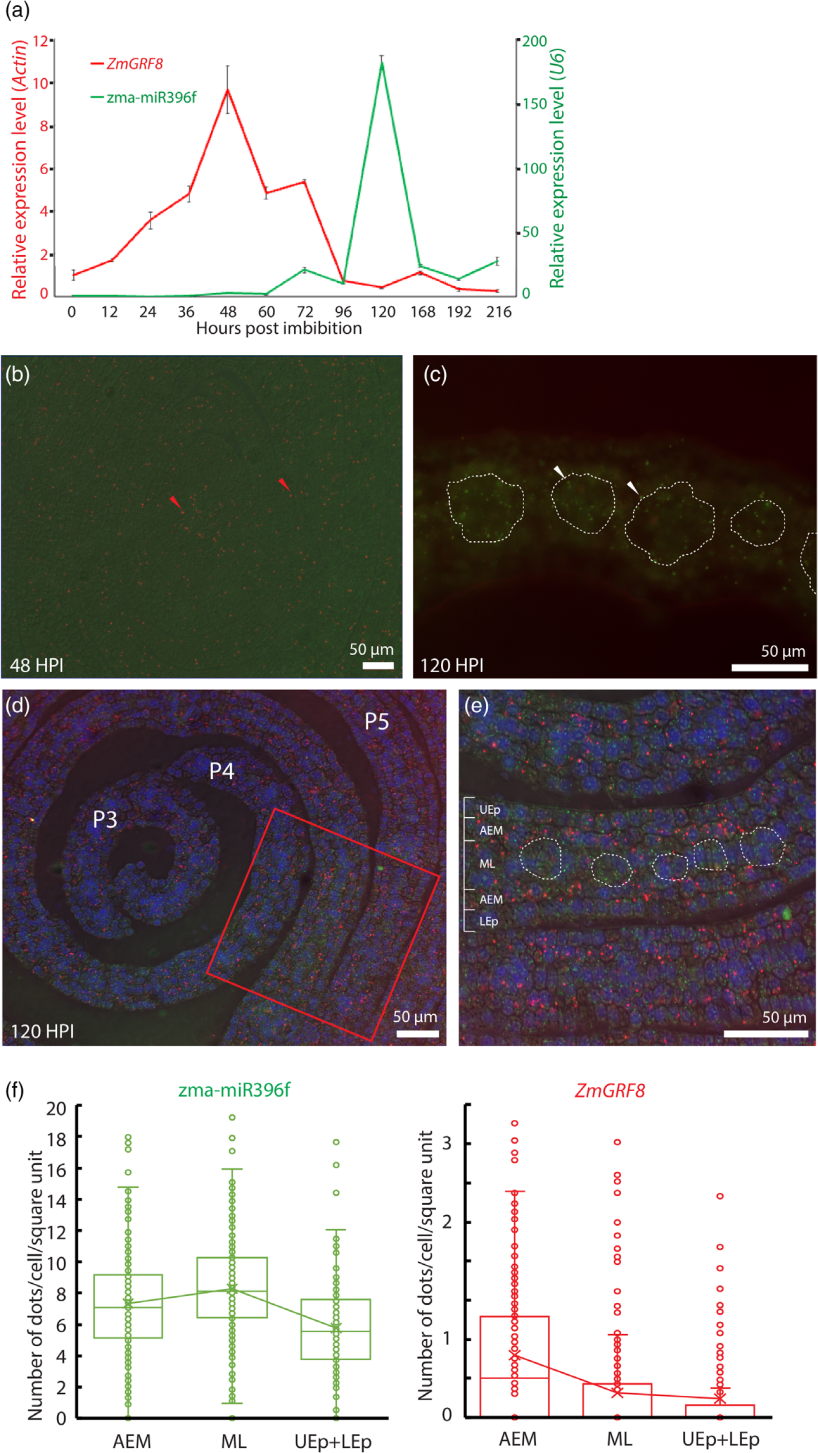
Relative expression levels and *in situ* simultaneous detection of zma‐miR396f and *ZmGRF8* molecules in maize developing leaf tissues. (a) Relative expression levels of zma‐miR396f and *ZmGRF8* after imbibition, assayed by qRT‐PCR. The lowest expression level of zma‐miR396f occurs before 60 h and the highest expression level occurs at 120 h post imbibition. In contrast, *ZmGRF8* shows the highest expression level at 48 h post imbibition. The expression level of zma‐miR319f or ZmGRF8 at 0 h is used as a reference for estimating their relative expression levels at different time points. (b) *In situ* detection of zma‐miR396f and *ZmGRF8* on maize developing leaves at 48 h post imbibition. Clear signals (red dots) of *ZmGRF8* are visible, whereas no signal of zma‐miR396f is detected. (c) *In situ* detection of zma‐miR396f on 1st maize developing leaves at 120 h post imbibition. Signals of zma‐miR396f (green dots) are visible, but no signal of *ZmGRF8* is detected. Overall, the *in situ* expression profiles of zma‐miR396f and *ZmGRF8* are consistent with the relative expression levels of zma‐miR396f and *ZmGRF8* in Figure 7a. White dashed‐lines enclose the vascular tissue. (d) *In situ* simultaneous detection of zma‐miR396f and *ZmGRF8* molecules in the base elongation zone of maize developing leaves at 120 h post imbibition. Signals of *ZmGRF8* (red dots) increase as leaves develop from P3 to P5, while signals of zma‐miR396f (green dots) are relatively abundant throughout these developmental stages. (e) The zoom‐in of the red rectangle in (d). *ZmGRF8* (red) is significantly accumulated in the mesophyll cells around vascular tissues (enclosed with white dashed‐lines) while zma‐miR396f (green) is abundant in epidermis and vascular tissues. (f) Box graphs showing the average numbers of dot signals of *ZmGRF8* and zma‐miR396f per cell in three tissue types, AEM, ML and epidermis (UEp + LEp) of maize embryonic leaves (see e). Cells of ML, where veins develop, have a relatively high expression of zma‐miR396f but a relatively low expression of *ZmGRF8*. In contrast, cells in AEM have a relatively low expression of zma‐miR396f, but a relatively high expression of *ZmGRF8*. In differentiated epidermis cells both zma‐miR396f and *ZmGRF8* show the lowest expression level. UEp: upper epidermis cells shown in (e). AEM, mesophyll cells below the epidermis cells; LEp, lower epidermis cells; ML, the middle layer of leaf.

As another example of multiplexed detection, we study the expression profiles of *ZmGRF8* and its potential miRNA regulator, zma‐miR396f, in developing maize leaves at 48 and 120 h post imbibition (Figure 7a). Our result shows that at 48 h post imbibition signals of *ZmGRF8* are detected in shoot apical meristems (SAM) and serially developing leaves (red dots in Figure 7b), but no signal of zma‐miR396f is visible (Figure 7b). In contrast, abundant signals of zma‐miR396f are detected in developing bundle sheaths within the middle layer of the 1st leaf (green dots), and no signal of *ZmGRF8* is seen at 120 h post imbibition (Figure 7c). These expression profiles are consistent with relative expression levels determined by qRT‐PCR (Figure 7a), suggesting that our method works properly for detecting both miRNA and mRNA transcripts in one run of detection probe labeling.

To further confirm the simultaneous detection of zma‐miR396f and *ZmGRF8*, we investigate the expression of these two genes at the base elongation region of the 1st maize developing leaf, where leaf cells are undergoing rapid cell division and cellular differentiation. Our results show that signals of zma‐miR396f and *ZmGRF8* can be simultaneously detected with different fluorophores. Both RCA products derived from zma‐miR396f and *ZmGRF8* molecules form bright colour round‐dot signals that are easily differentiated from plant tissue autofluorescence (Figure 7d,e). Moreover, *ZmGRF8* transcripts are abundant in mesophyll cells next to epidermis layers (AEM) and adjacent to veins (Figure 7e). In contrast, signals of zma‐miR396f are detected in all cellular types of leaves but are more abundant in developing vascular tissues (Figure 7e). Note that fewer signals of *ZmGRF8* are detectable in cells in which the signal of zma‐miR396f is abundant, showing differential cell‐specific expressions of zma‐miR396f and *ZmGRF8* (Figure 7e).

To verify this observation, we quantified the signals of zma‐miR396f and *ZmGRF8* within individual cells in different cellular tissues, including epidermis cells, a layer of mesophyll cells below the epidermis cells (AEM) and the middle layer of leaf (ML; Figure 7f). Our results show that cells of ML, where veins develop, have a relatively high expression of zma‐miR396f and a relatively low expression of *ZmGRF8* (Figure 7f). In contrast, cells in AEM have a relatively low expression of zma‐miR396f and a relatively high expression of *ZmGRF8*. These observations suggest that *zma‐miR396f* regulates *ZmGRF8* and are related to the differentiation of vascular bundles in the middle layer of a leaf. In differentiated epidermis cells, both *zma‐miR396f* and *ZmGRF8* show the lowest expression level among the three tissues (Figure 7f). In summary, our data suggest that zma‐miR396f regulates the expression of *ZmGRF8* during tissue development and differentiation of maize leaves.

## Discussion

In this study, we have developed a sensitive and versatile method for simultaneous detection of miRNA and mRNA molecules in isolated cells (Figure 3) and plant tissues (Figure 4). For miRNA hybridization, use of miRNA‐LNA probe achieves high sequence specificity for each target, reducing the chance of hybridization to off‐targets (Kierzek *et al*., 2005; Tolstrup *et al*., 2003), and the combination of the padlock probe with RCA, which amplifies detection signals, results in a high signal‐to‐noise ratio for the detection of miRNA and mRNA molecules at the single‐cell level (Gu *et al*., 2018). Our study demonstrated that this method can be used effectively for the simultaneous *in situ* detection of miRNA and mRNA molecules in fixed plant tissues even though plant materials show strong autofluorescence (Figures 3, 5 and 7). We also developed a protocol for *in vitro* miRNA detection to explore potential binding to off‐targets, providing a means to determine suitable hybridization conditions (Figures 2 and 3). Thus, discrimination of a single‐base difference between two miRNAs can be evaluated *in vitro*. Our method is also capable of detecting different expression levels of miRNA and its target mRNA molecules (Figures 3d, 5b and 7b,c).

It has been demonstrated that LNA modification sites at the 5′ end increases the efficiency of rolling circle amplification and thus increase the signal level of RCA with fluorescence‐tagged detection probes (Larsson *et al*., 2004, 2010). Based on this criterion, we examined the discrimination of base mismatches for the miRNA‐LNA probe we designed. As shown in a previous study (Tolstrup *et al*., 2003), different conformations and arrangements of LNA oligonucleotides show different levels of the discrimination for the mismatches between a target and a probe. Thus, users can modify the arrangement of LAN sites to optimize the specificity of probe hybridization and our dot blot approach can be used to verify the probe design. There is no strict restriction for miRNA‐LNA probe design, but the probe specificity is determined by the miRNA. In general, the length of 20 bases provides sufficient detection specificity even under a moderate hybridization condition. If a probe is longer than 20 bases, the melting temperature and hybridization condition of the probe may become relatively high and may cause defects on sections or samples.

The backbone region in the miRNA‐LNA padlock probe is the same as that of the mRNA padlock probe (Larsson *et al*., 2004, 2010). In general, there are four guidelines for the backbone region design: (i) in general, it is ~10 bases longer than the detection hybridization region, (ii) the GC content ranges from 50% to 60%, (iii) the numbers of the four nucleotides are similar and evenly distributed in a sequence, and (iv) the sequence does not form hairpin structures. An important condition is that the sequence is not highly similar to any sequence in the target genome. Under these criteria, this region can also be used for the compaction of the rolling circle amplification product to increase the signal integrity (Clausson *et al*., 2015).

To quantify signals of RCA within individual cellular boundary, it is essential to label and identify the cell boundary for automated signals quantification by an image software, such as the CellProfiler (Carpenter *et al*., 2006), or for manually counting. In plants, one can identify the cell wall or membrane. Many autofluorescent compounds, such as suberin, lignin ferulate and flavonoid, are bounded to cell wall, emitting weak to moderate blue fluorescence with UV excitation (Donaldson, 2020). In plants, cell walls in various tissue types, even in roots, comprise more or less the above compounds, and shine fluorescences under UV excitation, useful for cell boundary identification. Based on our experience, autofluorescence from cell walls is common in developing leaves, shoots and roots of rice and maize. It is also common in other species (Pegg *et al*., 2021). We have demonstrated this approach in plants for detecting osa‐miR156 and *OsSPL12* in rice leaves (Figure 6). In addition, the algorithm in the CellProfiler uses nucleus conformation stained with a fluorescence dye, such as DAPI, to explore and define cell boundary (Carpenter *et al*., 2006). We have utilized this approach to examine zma‐miR396f and *ZmGRF8* in different cellular types of embryonic leaves, and the identification of cell boundary is reliable. Alternative strategies are using antibodies specifically for cell wall components (Rydahl *et al*., 2018) or probes for cell membranes, such as BioTracker 400 Blue Cytoplasmic Membrane Dye (Merck Inc., Rahway, New Jersey, U.S.). In summary, several approaches for determining the cell boundary of plant tissues are available for signal quantification at the single‐cell level.

Our method facilitates the *in situ* characterization of the expression profiles of miRNAs and their target mRNAs for understanding the miRNA regulation of cellular development and differentiation at the single‐cell level. If a miRNA regulates a specific mRNA, the pair of miRNA and mRNA molecules could be expressed in the same cells or different cells depending on developmental stages. As cells develop and differentiate, the pair of miRNA and mRNA molecules will progressively show cell‐specific patterns. To test our method, we applied it to investigate the expression profiles of *ZmGRF8* and its potential miRNA regulator, zma‐miR396f, in developing maize leaves. The *in situ* hybridization data obtained indeed showed progressive specific expression profiles of zma‐miR396f and *ZmGRF8* in an opposite manner by cell types of developing maize leaves, providing evidence that zma‐miR396f negatively regulates the preferential spatial expression of *ZmGRF8* in bundle sheath cells of developing maize leaves (Figure 7).

Our approach can be readily applied to animal tissues. Most reagents and reaction buffers that we modified in this study for plant tissues had been used in mammalian tissues (Wu *et al*., 2019) or cell cultures (Weibrecht *et al*., 2013). Thus, with simple modifications, our method of miRNA and mRNA detection can be applied to animal tissues and paraffin‐embedded materials.

Another advantage is that our method can potentially be scaled up for multiplexed miRNA and/or mRNA detections using a conventional epifluorescence microscope by sequential imaging. Up to four different fluorophores, such as Alexa Fluor 488, Cyanine 3, Texas Red and Cyanine 5 (Hilscher *et al*., 2020), can in principle be simultaneously used for one run of imaging without cross‐talks among fluorophores. After one cycle of imaging, the section can be reused for the next cycle by stripping off the previous four detection oligos (Wu *et al*., 2019). Another four detection oligonucleotides for additional four targets can then be hybridized as in the previous cycle. In practice, five cycles of detection oligo hybridization and imaging can be achieved without significant signal degradation (Gyllborg *et al*., 2020; Hilscher *et al*., 2020). Thus, up to 20 miRNAs and/or mRNA targets can be spatially detected on the same tissue section. For multiplexed miRNA and/or mRNA detection, the comparison among genes could potentially be made. However, the comparison among genes is crude because the binding affinity of probes to target miRNAs could be different among probe‐miRNA pairs. If similar probe properties and hybridization affinities can be achieved and confirmed, “relative expression levels” can be interpreted on the same section. In general, this kind of comparison is crude.

In summary, we have developed a method that is capable of simultaneously detecting miRNA and mRNA molecules in plant tissues by combing LNA probes and padlock probes with RCA to achieve high sequence specificity and sequence‐based signal amplification. The method is robust, versatile and scalable to meet the emerging demands for detection of gene expression at the single‐cell level.

## Materials and methods

### Plant materials and growth condition

*Zea mays* cultivar White Crystal was used in this study. Seeds were imbibed in water for 1 day, and then planted at 28 °C in the dark for protoplast preparation or under a 16 h/8 h light–dark cycle for leaf section preparation.

The rice cultivar Tainung 67 (TNG67) was used as wild‐type accession for japonica rice in this study, and the T‐DNA activation mutant *miR156b/c_Act_* was obtained from the Taiwan Rice Insertional Mutants library (http://trim.sinica.edu.tw/). The rice seeds were sterilized by 2.5% sodium hypochlorite (NaClO) and then placed on wet paper towel at room temperature for a week. The sprouting seeds were moved to soil and grew in a walk‐in growth chamber at 28 °C under 16 h/8 h light–dark cycle.

### Plasmid construction and site direct mutagenesis

To *semi‐in‐vivo* verify the specificity of the miRNA‐LNA probe, we constructed the overexpression vectors of zma‐miR319b and point‐mutation versions at various sites, including b03, b05, b11 and b19 (Figures 2 and S1). A vector was constructed, in which it contains an inserted DNA fragment spanning from the 400 bp upstream of the 5′ end to the 400 bp downstream of the 3′ end of the zma‐miR319b precursor‐miRNA (zma‐miR319b), respectively. In brief, the zma‐miR319b sequences from the miRBase website (https://www.mirbase.org/) were blasted to the maize genome (Zm‐B73‐REFFERENCE‐NAM‐5.0) to select the sequence for cloning primer‐pair design. A cloning forward primer was designed around 400 bp upstream from the 5′ end of *zma‐MIR319b* and a reverse primer was located around 400 bp downstream to the 3′ end of *zma‐MIR319b*. The target DNA fragment was amplified by Phusion High‐Fidelity DNA Polymerase (Thermo Fisher Scientific, Waltham, MA) with respective cloning primer sets (Table S2), and cloned sequence of *zma‐MIR319b* of this cultivar was further verified by the Sanger sequencing (Data S1). The amplified product was further digested by *Not*I and *Kpn*I and cloned into the *p*AHC18_35G, which is a modified version of *p*AHC18 (Bruce *et al*., 1989).

We then constructed the overexpression vectors of zma‐miR319b point mutants (b03, b05, b11 and b19). We designed inverse point mutation primer pairs (Table S2) which overlapped 15 bases of the 3′ end, in which the desired mutation sites were designed at 8th base in overlapped region. The original zma‐miR319b overexpression vector was used as a template, and respective inverse point mutation primer pairs were combined with zma‐miR319b‐NotI‐IF‐F and zma‐miR319b‐KpnI‐IF‐R in PCR reaction (Table S2). zma‐miR319b‐*Not*I‐IF‐F was paired with zma‐miR319b‐3p‐b03‐IF‐R and zma‐miR319b‐3p‐b03‐IF‐F was paired with zma‐miR319b‐*Kpn*I‐IF‐R. For respective point mutants, two amplified PCR products were ligated into linear *p*AHC18_35G (digested by *Not*I and *Kpn*I) using GenBuilder™ Cloning Kit (GeneScript, Piscataway, NJ). After the first construction process was completed, the resulting plasmids were used as templates, and the previous steps were repeated until the point mutation of zma‐miR319b‐5p and zma‐miR319b‐3p in respective overexpression vectors were completed. All primers were listed in Table S2.

### Creation of *miR156b/c‐OE* transgenic rice

For construction of osa‐miR156b/c overexpression vector (*miR156b/c‐OE*), the 1039 bp fragment containing *osa‐MIR156b* and *osa‐MIR156c* was amplified by osa‐miR156b/c cloning primer sets (Table S2). The PCR product was digested with *EcoR*V and *Kpn*I, and then inserted into the maize ubiquitin promoter in *p*AHC18 vector (Bruce *et al*., 1989). This plasmid was further linearized by *Hind*III and ligated into *p*CAMBIA‐1301 to form transformation vector. The *miR156b/c‐OE* rice transformation was conducted via *Agrobacterium*‐mediated transformation as previous described (Hiei *et al*., 1997).

### Plant protoplast preparation and transfection

Mesophyll protoplasts were isolated from fully expanded leaves of 1‐week‐old etiolated maize seedling as previously described (Chang *et al*., 2012). Isolated protoplasts were adjusted to 5 × 10^5^ cells/mL and transformed with a ratio of 10 μg plasmid (the miRNA overexpression vector) per 10^5^ cells (Chang *et al*., 2019). Transfected protoplasts were incubated in Falcon culture plates at 26 °C in the dark for 14 h, and then harvested by centrifugation at 150 *g* for 2 min for either RNA extraction or *in situ* padlock probe with rolling circle amplification (RCA) analysis.

### Plant section preparation

Developing leaves of maize seedlings at specific hours post imbibition were dissected and fixed in a mixture of 75% ethanol and glacial acetic acid at the ratio 3:1 at 4 °C for 12 h. After three washes of phosphate‐buffered saline (PBS), fixed samples were infiltrated with a serial gradient of sucrose from 5% to 33% in PBS, and then embedded in optimal cutting temperature (OCT) compound (Leica co., Wetzlar, Germany). Cross sections of 14 μm thickness were obtained using a Leica Cryostat CM1950 (Leica co., Wetzlar, Germany) for *in situ* detection of miRNA and mRNA molecules.

### *In vitro* miRNA detection

To study the binding specificity of our miRNA‐LNA probe, we used a modified dot blot to detect synthesized DNA oligonucleotides of 20 bases. Synthesized short oligonucleotides of 20 bases were incubated at 95 °C for 5 min and immediately chilled on ice for 5 min. Two *p*moles of synthesized oligonucleotides were applied on Amersham Hybond‐N+ membrane (Cytiva, Marlborough, MA) and fixed by using UV crosslinking (120 000 μJ/cm^2^). The membrane was washed with wash buffer (1× PBS pH 7.4, 0.05% Tween® 20) for 5 min at room temperature and then incubated in the blocking buffer (50% formamide, 5× SSC, 0.5 μg/μL tRNA, 1× Denhardt's solution) for 1 h at 10 °C below predicted melting temperature of miRNA‐LNA probe. Ten *p*moles of the miRNA‐LNA probe were then applied to the membranes in secured incubation chamber and incubated for an hour (in 50% formamide, 5× SSC, 0.5 μg/μL tRNA, 1× Denhardt's solution). After washes with 0.1× SSC five times followed by one wash with 2x SSC, membranes were washed twice with 0.05% Tween 20 in PBS.

The 5′‐ and 3′‐ends of the corresponding padlock probes (0.1 m) were then hybridized to the 3′ single‐stranded portion of the miRNA‐LNA probe. The gap remaining at the hybridized 3′‐ and 5′‐ends of padlock probes was annealed with *Taq* DNA Ligase (0.5 U/μL, with 0.2 μg/μL BSA, 0.05 m KCl, 20% formamide). Circled padlock probes were then served as templates for subsequent RCA amplification. After RCA amplification in a mixture (1 U/μL Phi29 DNA polymerase, 0.25 mm dNTP, 0.25 mm biotin‐dUTP, 0.2 μg/μL BSA, 5% glycerol), the membrane was incubated in the blocking buffer [1% Blocking Reagent (Roche, Basel, Switzerland) in 1× PBS pH 7.4, 0.05% Tween® 20, 0.1% SDS] with the Streptavidin‐HRP Conjugate (Cytiva, Marlborough, MA) at the ratio of 1:2000 for 1 h at room temperature with gentle shaking. Then, the membrane was washed with wash buffer five times. Immobilon Western Chemiluminescent HRP Substrate (Millipore, Burlington, MA) was then used for chemiluminescent detection, and the chemiluminescent signals were visualized by the BioSpectrum imaging system (UVP, Upland, CA).

### miRNA molecule detection on maize mesophyll protoplast

Transformed protoplasts were collected and fixed in a mixture of 50% ethanol and glacial acetic acid at the ratio 3:1 at 4 °C for 20 min, followed by the dehydration of a serial gradual ethanol from 50% to 100%. Drops of 100% EtOH containing fixed transformed protoplasts were applied on ThermoFisher Superfrost Plus microscope slides, and then air‐dried. Slides were stored in −80 °C freezer for further *in situ* detection.

### *In situ* detection of miRNA molecules

Slides with maize developing leaf sections were permeabilized in 0.05 U/μL proteinase K at 37 °C for 5 min, followed by washes of DEPC‐PBS twice. Two *p*moles of miRNA‐LNA probe (Table S1) were then hybridized to target miRNA molecules on sections for 1 h (in 50% formamide, 5× SSC, 0.5 μg/μL tRNA, 1× Denhardt's solution) at 10 °C below the predicted melting temperature of miRNA LNA probe. After washes of 0.1× SSC five times followed by one wash of 2× SSC, sections were washed twice with 0.05% Tween 20 in PBS. The 5′ and 3′ ends of the corresponding 0.1 m padlock probes (Table S1) were then hybridized to the single‐stranded portion of the LNA probe. The gap at the hybridized 3′ and 5′ ends of padlock probes was annealed with *Taq* DNA Ligase (0.5 U/μL, with 0.4 U/μL RNase H, 1 U/μL Ribolock RNase Inhibitor, 0.2 μg/μL BSA, 0.05 m KCl, 20% formamide). Circled padlock probes were then used as templates for subsequent RCA amplification. After RCA amplification in a mixture (1 U/μL Phi29 DNA polymerase, 0.25 mM dNTP, 0.2 μg/μL BSA, 5% glycerol), 0.1 μM specific detection oligonucleotides (1× SSC, 20% (V/V) formamide; Table S1) were hybridized to the RCA molecules derived from miRNA. Images were taken using a Zeiss Z1 microscope with proper filter sets.

### Simultaneous *in situ* detection of mRNA and miRNA molecules

Slides with maize developing leaf sections were permeabilized in 0.05 U/μL proteinase K at 37 °C for 5 min, followed by washes of DEPC‐PBS twice. Two *p*moles of the miRNA‐LNA probe were then hybridized to target miRNA molecules on sections for an hour at 10 °C below the predicted melting temperature of miRNA LNA probe (in 50% formamide, 5× SSC, 0.5 μg/μL tRNA, 1× Denhardt's solution). After washes of 0.1× SSC five times followed by one wash of 2× SSC, sections were washed with 0.05% Tween 20 in PBS twice more. Then, reverse transcription of the target mRNA with the LNA primer was performed at 37 °C for 3 h. Subsequently, 0.1 μm padlock probes of both the miRNA and its target mRNA were hybridized and ligated as described above. The two types of detection oligonucleotides were hybridized to respective RCA products derived from specific padlock probes of mRNA and miRNA. Images were taken using a Zeiss Z1 microscope with proper filter sets. The detailed procedure is available on the protocols.io (https://www.protocols.io/blind/CE5D27FF262A11ED9BA60A58A9FEAC02).

### Quantification of RCA signals

*In situ* mRNA abundance was quantified by counting independent, fluorescent signals on a Zeiss fluorescence microscope Z1 equipped with a Zeiss Axiocam Mrm 1.4 MP monochrome CCD digital camera and corresponding filter setting. Fluorescent dyes were chosen such that miRNA or mRNA abundance could be investigated simultaneously on transformed mesophyll protoplasts. Quantification of signal position, intensity and cumulative number of RCA signals was automated using a Cellprofiler ver. 2.4.0rc1 pipeline (Carpenter *et al*., 2006). The basic steps of the pipeline were segmentation of the protoplast outline, detection of fluorescent signals, and detection of autofluorescence. The outline of the protoplast was extracted by enhancing the edges followed by morphological operations. The outline of the cells on sections was extracted by enhancing the autofluorescence in the DAPI channel. The fluorescent signals were segmented using an adaptive local thresholding method based on ellipse fit (Ranefall *et al*., 2016). All scripts are available in the supplement scripts and codes.

### RNA extraction and qRT‐PCR

Total RNA from seedlings post imbibition was extracted with TRizol reagent (Invitrogen, Carlsbad, CA) following a previous procedure (Liu *et al*., 2013). For RT reaction, 20 ng total RNA with mRNA or miRNA specific stem‐loop RT primers (Table S2) were subjected to a RT master mix that contained 250 μm dNTP, 1× First‐Strand buffer, 10 mm DTT, 4 units of RNaseOUT™ and 50 units of SuperScript™ IV Reverse Transcriptase in 20 μL reaction volume (Invitrogen, Carlsbad, CA). The RT reaction was performed as previously described (Varkonyi‐Gasic *et al*., 2007). The qRT‐PCR analysis was conducted as previously described (Chang *et al*., 2019) using 1 μL of RT product with the respective mRNA or miRNA‐specific forward primer and a reverse primer. The expression levels were normalized using the threshold cycle (*C*_t_) values obtained for the *Zm‐ACT* or U6 reference gene, and the $2^{-\Delta \Delta C_{t}}$ method was used for relative quantification (Livak and Schmittgen, 2001).

## Conflict of interest

The authors have no conflict of interest to declare.

## Author contributions

C.‐C.W. and W.‐H.L. conceived the study. W.‐H.L., M.S.B.K. and L.‐J.C. advised the study. C.‐C.W. and K.‐T.H. designed the experiments. C.‐C.W., K.‐T.H., S.‐Y.Y. and Y.‐T.L. conducted the experiments. C.‐C.W and W.‐H.L. wrote the first draft. All authors participated in writing the paper.

## Supporting information


**Data S1** The sequence of zma‐MIR319b of *Zea mays* cultivar “white crystal”.

**Figure S1** Transient expression vector and modified dot blot hybridization of zma‐miR319b with various point mutations.
**Figure S2** Transient overexpression of zma‐miR319b‐3p with a point mutation in mesophyll protoplasts of leaves of a 1‐week‐old maize etiolated seedling.
**Figure S3** Signal quantification of transient overexpression of zma‐miR319b‐3p in mesophyll protoplasts of maize leaves of a 1‐week‐old etiolated seedling by the CellProfiler pipeline.
**Figure S4** Dynamic expression of osa‐miR156 in TNG67 developing leaves.
**Figure S5** Dynamic expression of *OsSPLs* in TNG67 developing leaves.
**Figure S6** A biological replicate of Figure 5.
**Figure S7** Signal quantification of osa‐miR156 and *OsSPL12* in a young leaf of the osa‐miR156b/c overexpression transgenic rice (*miR156b/c‐OE*) by the CellProfiler pipeline.

**Table S1** LNA probes, padlock probes and detection oligonucleotides for *in situ* detection of miRNA and mRNA.
**Table S2** Primer sequences used in plasmid construction, qRT‐PCR, and transgenic rice line genotyping.
