# Supplementary material for: Simultaneous detection of miRNA and mRNA at the single‐cell level in plant tissues
Source: Plant Biotechnol J. 2022 Oct 20;21(1):136–49. doi: 10.1111/pbi.13931 (PMC9829392; doi:10.1111/pbi.13931)
Supplement: Supplementary file 1 — Data S1 The sequence of zma‐MIR319b of Zea mays cultivar “white crystal”. [file PBI-21-136-s002.docx]

Supplementary data 1. The sequence of zma-MIR319b of *Zea mays* cultivar “white crystal”. The bases highlight in color (yellow+green+blue) is pre-zma-miR319b. The region in green is zma-miR319b-5p and that in blue is zma-miR319b-3p.

GTCATGGTTAGTTAGTTGCGACGATGGCTGGATGGAAGAGAGCGTCCTTCAGTCCACTCAGGGGCGGTGCTAGGGTCGAATTAGCTGCCGACTCATTCACCCACATGCCAAGCAAACAGCCATGGAAACCAGCTTTGCAGATGAGTGAATGAAGCGGGAGGTAAAAGCTTCGATCTCGCACCGTCTTTGCTTGGACTGAAGGGTGCTCCCTCTGTCCCTCCTCCCTCCTTGTTTACCTTTATCGATCCATGCATCAGCTTCGTCATATCTCATGGTTTTACTAG
