# Supplementary material for: Simultaneous detection of miRNA and mRNA at the single‐cell level in plant tissues
Source: Plant Biotechnol J. 2022 Oct 20;21(1):136–49. doi: 10.1111/pbi.13931 (PMC9829392; doi:10.1111/pbi.13931)
Supplement: Supplementary file 2 — Figure S1 Transient expression vector and modified dot blot hybridization of zma‐miR319b with various point mutations. Figure S2 Transient overexpression of zma‐miR319b‐3p with a point mutation in mesophyll protoplasts of leaves of a 1‐week‐old maize etiolated seedling. Figure S3 Signal quantification of transient overexpression of zma‐miR319b‐3p in mesophyll protoplasts of maize leaves of a 1‐week‐old etiolated seedling by the CellProfiler pipeline. Figure S4 Dynamic expression of osa‐miR156 in TNG67 developing leaves. Figure S5 Dynamic expression of OsSPLs in TNG67 developing leaves. Figure S6 A biological replicate of Figure 5. Figure S7 Signal quantification of osa‐miR156 and OsSPL12 in a young leaf of the osa‐miR156b/c overexpression transgenic rice (miR156b/c‐OE) by the CellProfiler pipeline. [file PBI-21-136-s003.pdf]

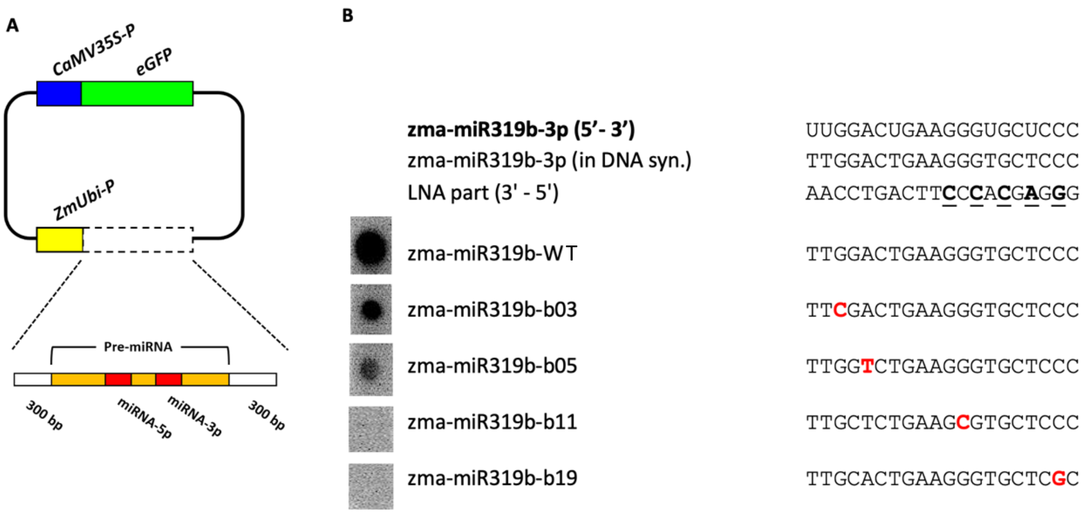

Supplementary Figure 1. Transient expression vector and modified dot blot hybridization of zma-miR319b with various point mutations. (A) A diagram showing the miRNA overexpression vector. (B) zma-miR319b-3p sequences each with a point mutation (in red) for transient overexpression vectors, including zma-miR319b-WT, b03, b05, b11 and b19. Each dot blot at the left shows the signal intensity of a wild type or a mutant sequence on modified dot blot (see Figure 2).

A.

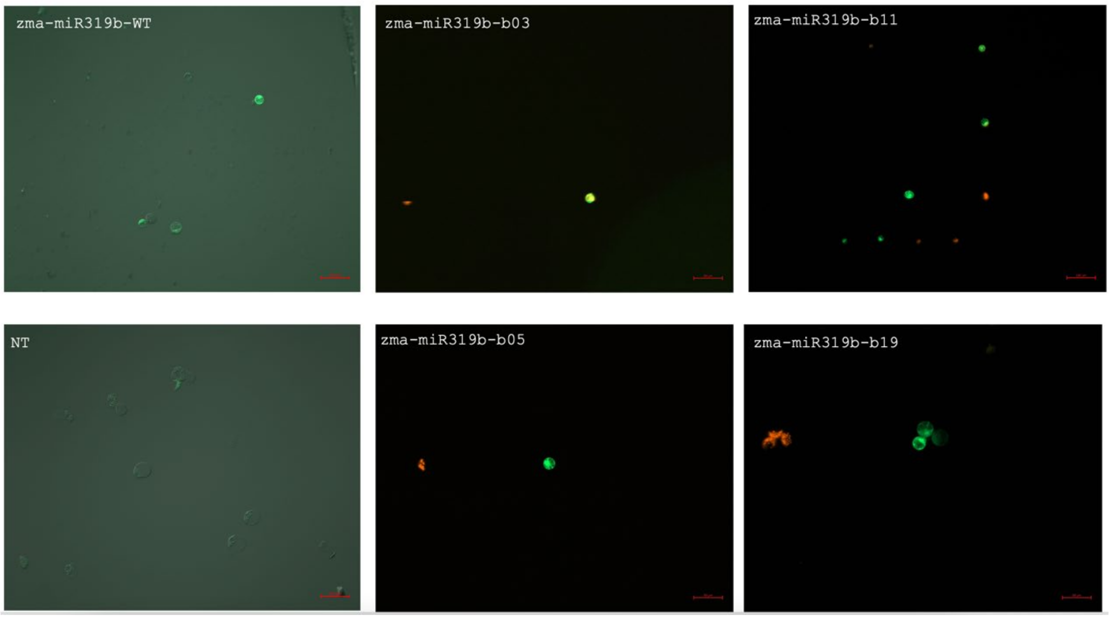

B.

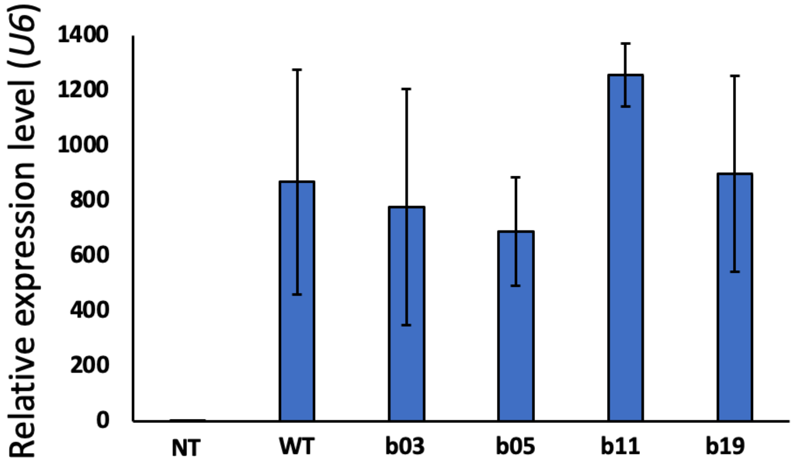

Supplementary Figure 2. Transient overexpression of zma-miR319b-3p with a point mutation in mesophyll protoplasts of leaves of a one-week-old maize etiolated seedling. (A) Signals of green fluorescent protein (GFP) indicate successful transformation of an overexpression vector carrying a zma-miR319b sequence with a point mutation in maize protoplasts. NT denotes "non-transformed" and serves as a negative control. (B) Comparison of relative expression levels of zma-miRNA319b-3p sequences each with a point mutation in transformed protoplasts. The expression levels were determined using qRT-PCR.

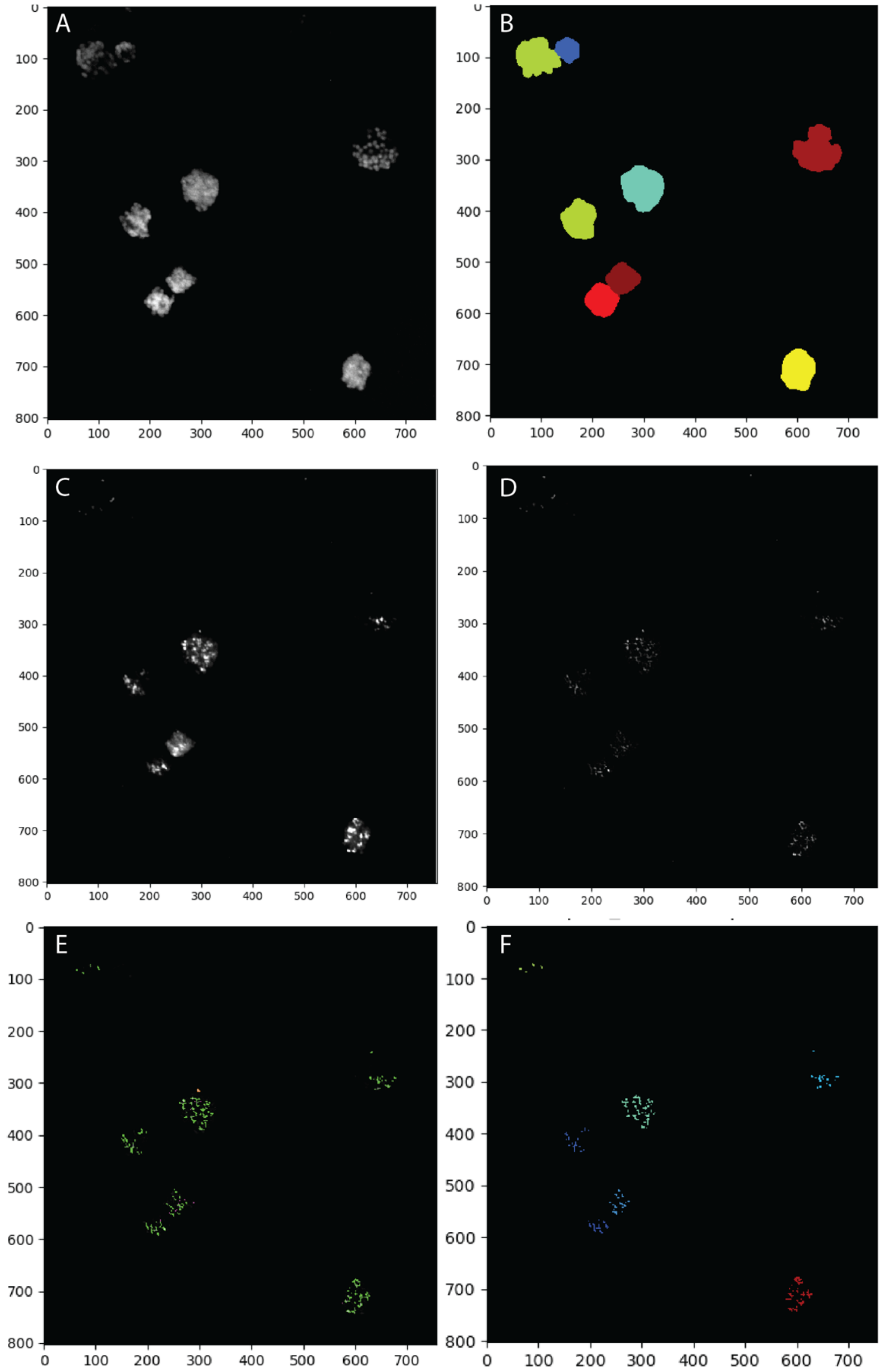

Supplementary Figure 3. Signal quantification of transient overexpression of zma-miR319b-3p in mesophyll protoplasts of leaves of a one-week-old maize etiolated seedling by the CellProfiler pipeline. (A) Autofluorescence of transformed protoplasts

in the cyanine 3 channel, showing the individual cell boundary. (B) Individual cells are identified and colored by different colors. (C) Signals of zma-miR319b-3p in the cyanine 5 channel. (D) Enhanced single molecules of zma-miR319b-3p. (E) Identified signals of zma-miR319b-3p. (F) Identified signals of zma-miR319b-3p in cells. The X and Y coordinates are in terms of pixels.

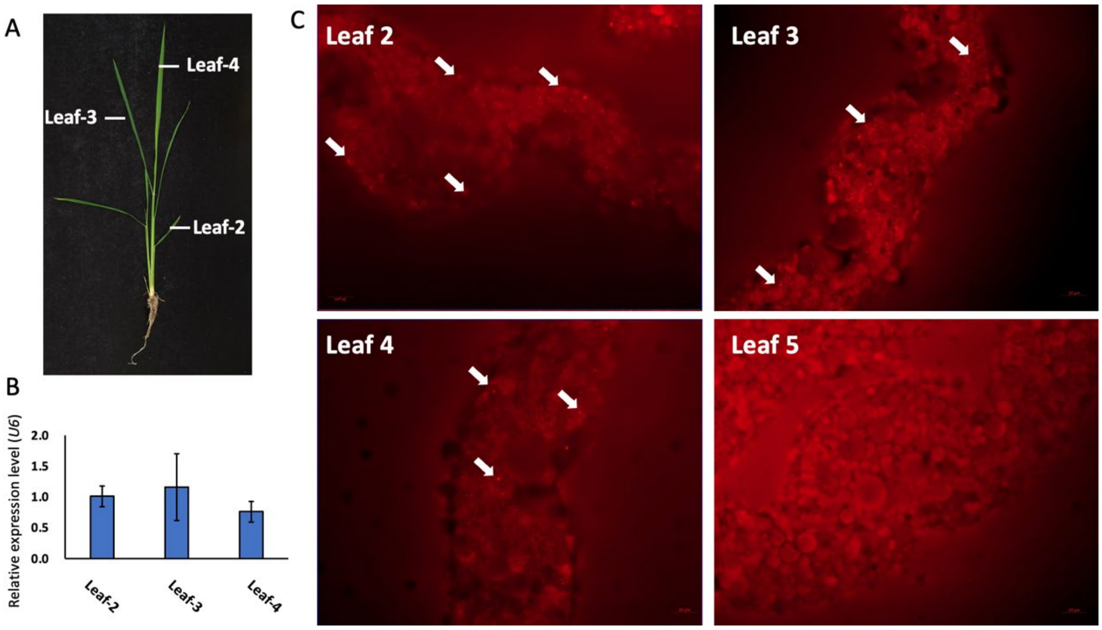

Supplementary Figure 4. Dynamic expression of osa-miR156 in TNG67 developing leaves. (A) Rice seedlings showing serial developing leaves, including the 2nd, 3rd, 4th and 5th leaves; the 5th leaf is the youngest visible leaf. (B) Relative expression levels of osa-miR156 in developing leaves determined by qRT-PCR. (C) *In situ* detection of osa-miR156 (some spots pointed by white arrows) in the 2nd to the 5th leaf, showing expression levels of osa-miR156 similar to those quantified by qRT-PCR in (B).

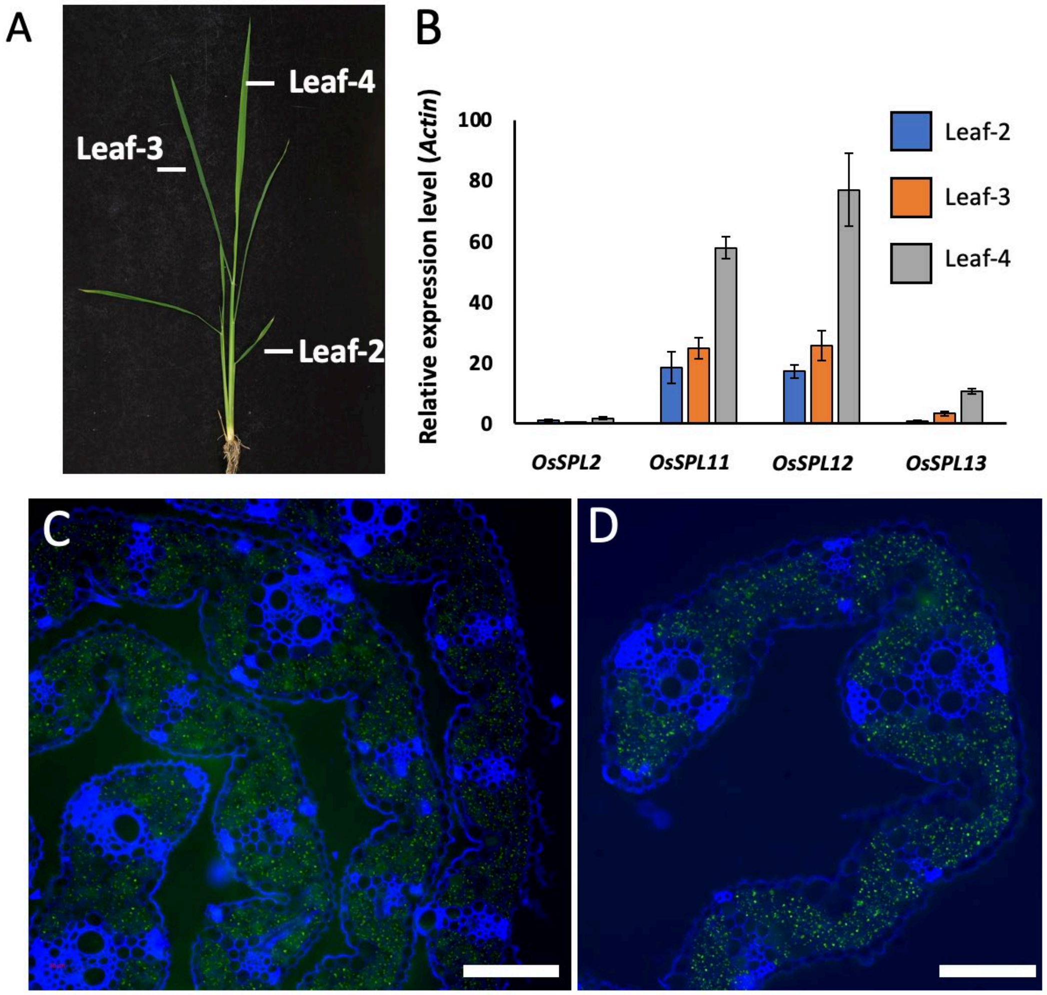

Supplementary Figure 5. Dynamic expression of *OsSPLs* in TNG67 developing leaves. (A) Rice seedlings showing serial developing leaves, including the 2nd, 3rd, 4th and 5th leaves; the 5th leaf is the youngest visible leaf. (B) Relative expression levels of *OsSPLs,* including *OsSPL2*, *OsSPL11*, *OsSPL12* and *OsSPL13*, in serial developing rice leaves. The expression levels of *OsSPL11*, *OsSPL12,* and *OsSPL13* decrease as leaves become mature. In addition, *OsSPL11* and *OsSPL12* are relatively highly expressed, compared to *OsSPL13*. (C) *In situ* detection of *OsSPL11* (green dots) in the 5th leaf. (D) *In situ* detection of *OsSPL12* (green dots) in the 4th leaf. Blue is autofluorescence of cell wall. Bar=50 μm.

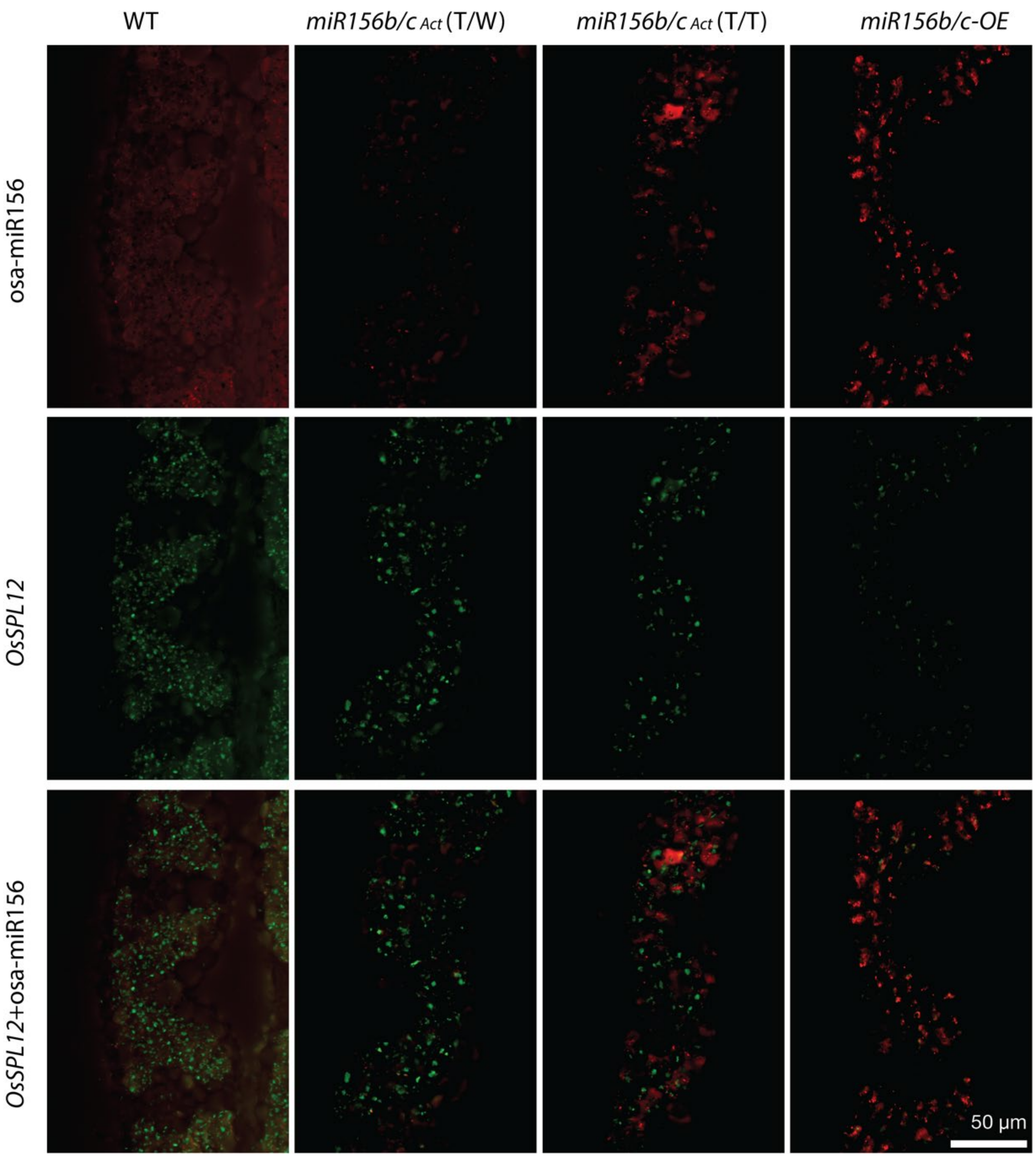

Supplementary Figure 6. A biological replicate of Figure 5. It shows the simultaneous *in situ* detection of osa-miR156 and *OsSPL12* on the same leaf sections of WT, *miR156b/c*$_{Act}$ mutants and *miR156b/c-OE*. The three rows of images show the *in situ* patterns of individual signals of osa-miR156 and *OsSPL12* (the first two rows) and the combined signals of *OsSPL12*and osa-miR156 (the third row) on the same section.

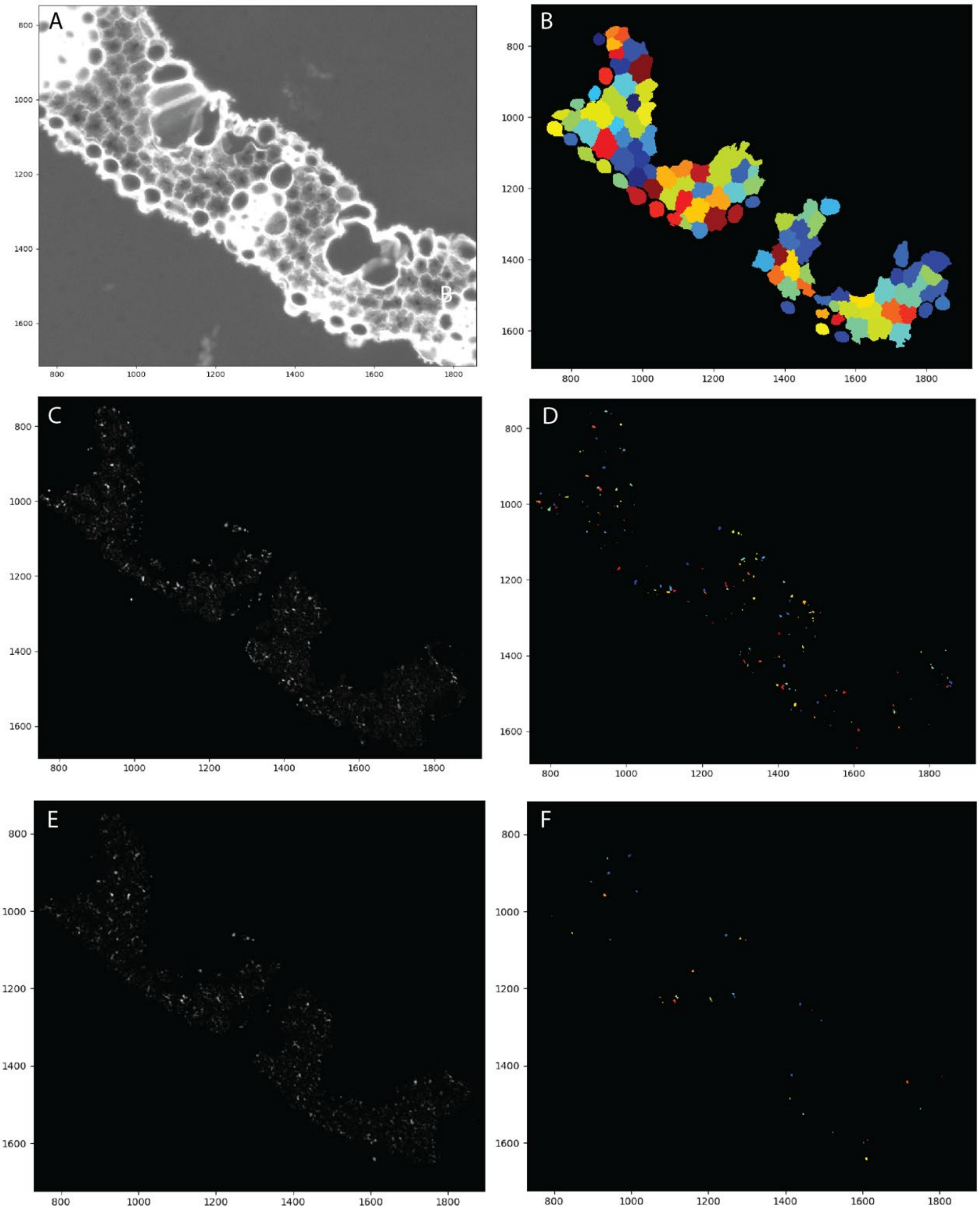

Supplementary Figure 7. Signal quantification of osa-miR156 and *OsSPL12* by the CellProfiler pipeline in a young leaf of the transgenic rice overexpressing osa-miR156b/c (*miR156b/c-OE*). (A) Autofluorescence of a leaf section in the DAPI channel, showing the cell boundary. (B) Cell identification in a leaf section by the CellProfiler pipeline. Individual cells are labeled by different colors. Cells of vascular bundles and bulliform cells are excluded. (C) Enhanced images in the cyanine 5 channel, showing osa-miR156 signals. (D) Identified signals of osa-miR156 in different cells are displayed by different colors. (E) Enhanced images in the cyanine 3

channel, showing *OsSPL12* signals. (F) Identified signals of *OsSPL12* in different cells are displayed by different colors. The X and Y coordinates are in terms of pixels.
