# Supplementary material for: Simultaneous detection of miRNA and mRNA at the single‐cell level in plant tissues
Source: Plant Biotechnol J. 2022 Oct 20;21(1):136–49. doi: 10.1111/pbi.13931 (PMC9829392; doi:10.1111/pbi.13931)
Supplement: Supplementary file 3 — Table S1 LNA probes, padlock probes and detection oligonucleotides for in situ detection of miRNA and mRNA. Table S2 Primer sequences used in plasmid construction, qRT‐PCR, and transgenic rice line genotyping. [file PBI-21-136-s001.pdf]

Supplementary Table 1. LNA probes, padlock probes and detection oligonucleotides for *in situ* detection of miRNA and mRNA. The bases in the lower case in a LNA probe are LNA modified sites and based in the bold are the padlock probe hybridization site. In a padlock probe, the bases in red are the hybridization site of detection oligonucleotide, the bases in blue are the 5’ and 3’ hybridization tails, and the bases in green are the backbone region. Fluorescent dyes for detection oligonucleotides: Cy3 = Cyanines 3, Cy5 = Cyanines 5

**Primer or probe**	**Sequence**
**LNA primer or porbe**	
ZmGRF8 LNA	5’/TcTtGaCgGtATTCCATTCGG
zma-miR396f-5p LNA	5’/TtCcAcAgCtTTCTTGAACTT**CGACAGTCCCAGTGTACCCACAAACCGTGAT**
ZmTCP44 LNA	5’/GaCtGcGaAtTGGACTGAAG
zma-miR319b-3p LNA	5’/GgGaGcAcCcTTCAGTCCAA**CACGCCGAAAGCATAAACGACGAGCAGATAT**
OsSPL11 LNA	5’/TcCaTtGtGaACTGAAACAC
OsSPL12 LNA	5’/CgTaAaAcAtCGTGGCGAGC
osa-miR156b-5p LNA	5’/GtGCtCAcTCtCTTCTGTCA**ATATCTGCTCGTCGTAGTTCTAACACTTTC**
**Padlock probes**	
Padlock probe *ZmGRF8*	5'/Phos/TCCAGATCGCCGAATAGCGATCTGCGAGACCGTATGGCTGTGCTTGGAGTCAATCGCAAGTAGGAGACTTCAACACCAGC
Padlock probe zma-miR396f-5p	5’/Phos/CACTGGGACTGTCGCTAGTGCTGGATGATCGTCCGGCTGTGCTTGGAGTCAATCGCAAGTAGGACACGGTTTGTGGGTA
Padlock probe *ZmTCP44*	5’/Phos/TAGGGGGACCCTTCACTAGTGCTGGATGATCGTCCGGCTGTGCTTGGAGTCAATCGCAAGTAGGATTTCTTGGGTGCCAG
Padlock probe zma-miR319b-3p	5’/Phos/TATGCTTTCGGCGTGAGCGATCTGCGAGACCGTATGGCTGTGCTTGGAGTCAATCGCAAGTAGGAATATCTGCTCGTCGTT
Padlock probe *OsSPL11*	5’/Phos/AGAGCCTCAGGTGTTCTAGTGCTGGATGATCGTCCGGCTGTGCTTGGAGTCAATCGCAAGTAGGAGACAACATCAAGCAGT
Padlock probe *OsSPL12*	5’/Phos/TTCATCAAGGCTCGCCTAGTGCTGGATGATCGTCCGGCTGTGCTTGGAGTCAATCGCAAGTAGGAAGGCAATTTCATTTGG
Padlock probe osa-miR156b-5p	5’Phos/ACGACGAGCAGATATAGCGATCTGCGAGACCGTATGGCTGTGCTTGGAGTCAATCGCAAGTAGGAAGAAAGTGTTAGAACT
**Detection oligonucleotide**	

Detection oligonucleotide 1852	5’/Cy3/CTAGTGCTGGATGATCGTCC
Detection oligonucleotide 1854	5'/Cy5/AGCGATCTGCGAGACCGTAT

Supplementary Table 2. Primer sequences used in plasmid construction, qRT-PCR, and transgenic rice line genotyping. Restriction enzyme cutting sequences are underlined. Point mutation sites of zma-miR319b are in red.

Primer	Sequence
**Plasmid construction**	
zma-miR319b-NotI-OE-F	ATGCGGCCGCGGAATACGCTGCCTGCTGTG
zma-miR319b-KpnI-OE-R	ATGGTACCCTCCTTGATGTATTGCCCTTGG
zma-miR319b-NotI-IF-F	AGTTCTAGAGCGGCCGCGGAATACGCTGCCTGCTGTG
zma-miR319b-KpnI-IF-R	CGGATCCCCGGGTACCCTCCTTGATGTATTGCCCTTGG
zma-miR319b-5p-b3-IF-F	TTCAGTCGACTCAGGGGCGGTGCTAGGG
zma-miR319b-5p-b3-IF-R	CCTGAGTCGACTGAAGGACGCTCTCTTCCATCCA
zma-miR319-5p-b5-IF-F	CCTTCAGACCACTCAGGGGCGGTGCTAGG
zma-miR319b-5p-b5-IF-R	TGAGTGGTCTGAAGGACGCTCTCTTCCATCCA
zma-miR319b-5p-b11-IF-F	GAGCGTCGTTCAGTCCACTCAGGGGCGG
zma-miR319b-5p-b11-IF-R	GACTGAACGACGCTCTCTTCCATCCAGCCAT
zma-miR319b-5p-b19-IF-F	TGGAAGACAGCGTCCTTCAGTCCACTCAGGG
zma-miR319b-5p-b19-IF-R	GGACGCTGTCTTCCATCCAGCCATCGTCG
zma-miR319b-3p-b3-IF-F	TTTGCTTCGACTGAAGGGTGCTCCCTCTGTCC

zma-miR319b-3p-b3-IF-R	TTCAGTCGAAGCAAAGACGGTGCGAGAT
zma-miR319b-3p-b5-IF-F	TGCTTGGTCTGAAGGGTGCTCCCTCTGTCC
zma-miR319b-3p-b5-IF-R	CCTTCAGACCAAGCAAAGACGGTGCGAGAT
zma-miR319b-3p-b11-IF-F	GACTGAACGGTGCTCCCTCTGTCCCTCC
zma-miR319b-3p-b11-IF-R	GAGCACCGTTCAGTCCAAGCAAAGACGGTGCG
zma-miR319b-3p-b19-IF-F	GGGTGCTGCCTCTGTCCCTCCTCCCTCC
zma-miR319b-3P-b19-IF-R	ACAGAGGCAGCACCCTTCAGTCCAAGCAAA
osa-MIR156b/c-EcoRV-OE-F	CAAGCGATATCAGGAGGAAGAGAGGGGTGAG
osa-MIR156b/c-KpnI-OE-R	CAGGTACCCAGTAAGCACCAGCACATACA
p1302_35Sp-IF-F	GGCCAGTGCCAAGCTTGGTGGAGCACGACACACTTG
p1302_NOSt-IF-R	GCAGGCATGCAAGCTTTTCCCGATCTAGTAACATAGATGAC
**qRT-PCR**	
zma-MIR319b-qF	GTCATGGTTAGTTAGTTGCGACGAT
zma-MIR319b-qR	AGGTAAACAAGGAGGGAGGAGG
OsAct-qF	TTCCAACAGATGTGGATATCTAAGG
OsAct-qR	AGTACCGACACACGCCCAAC
osa-miR156-qF	GCGCCCTGACAGAAGAGAGT
osa-miR156-SL-R	GTCGTATCCAGTGCAGGGTCCGAGGTATTCGCACTGGATACGACGTGCTC

OsSPL2-qF	TGCGAGGCGCACTCCAAGA
OsSPL2-qR	CGAGGTGAATCTTGCCGCT
OsSPL11-qF	GCAGTGCAGCAGGTTCCAC
OsSPL11-qR	GTCAGATGTCCACGAGGAAC
OsSPL12-qF	CAGTGTAGTCGGTTTCATGGT
OsSPL12-qR	TCCCAGGTTGTCACATGAAGA
OsSPL13-qF	CTGCTGGCGCGTCTTCTAG
OsSPL13-qR	GCTCATGGAACCGGCTGCA
U6-qF	CGATAAAATTGGAACGATACAGA
U6-qR	ATTTGGACCATTTCTCGATTTGT
zma-miR319b-3p-qF	GCGCCCTTGGACTGAAGGGT
Zma-miR319b-3p-SL-R	GTCGTATCCAGTGCAGGGTCCGAGGTATTCGCACTGGATACGACGG GAGC
Universal-SL-R	GCAGGGTCCGAGGTATTCGCAC
**Genotyping**	
miR156b/c $_{Act}$-F	TCCTCCACAGCTCACCATCCAA
miR156b/c $_{Act}$-R	TGCACACCTTCTTCTGACGAC
LB	CGTCCGCAATGTGTTATTAAGTT
